# Mexican American Cultural Values Scale: Considerations for college students

**DOI:** 10.1371/journal.pone.0354954

**Published:** 2026-09-01

**Authors:** Anjelica Martinez, Ashley Bautista, Alejandro L. Vázquez, German Cadenas, Amanda Venta

**Affiliations:** 1 Kinder Institute for Urban Research, Rice University, Houston, Texas, United Sates of America; 2 Department of Psychology, University of Houston, Houston, Texas, United States of America; 3 Department of Psychology & Neuroscience, University of Tennessee, Knoxville, Knoxville, Tennessee, United States of America; 4 Department of Clinical Psychoogy, Rutgers University, New Brunswick, New Jersey, United States of America; Instituto Tecnologico Autonomo de Mexico, MEXICO

## Abstract

The Mexican American Cultural Values Scale is among the most widely utilized scales to examine Latinx cultural values. Originally conceptualized to assess Mexican American values among adolescent Mexican Americans and their caregivers, the MACVS continues to be relied on for examining Latinx college student outcomes, leading to questions over utilization of the MACVS for this sample. This psychometric study used extant data from undergraduates enrolled in Hispanic-serving institutions (*n* = 495; 341 Mexican American) to test the internal consistency and MACVS model fit capturing Mexican American Traditional Values (*Familism-Obligation, Familism-Obligations, Familism-Referent, Respect, Religion,* *and* *Traditional Gender Roles)* and Mainstream Values (*Material Success, Independence & Self-Reliance,* and *Competition & Personal Achievement)*. Confirmatory factor analyses of the higher-order correlated model and alternative models indicated misfit for Mexican American college students, raising concerns regarding its use for this population: *X*^*2*^ (1176) = 3951.72, CFI = .73, TLI = .71, RMSEA = 0.08, SRMR = 0.18. Exploratory analyses intended to broaden the MACVS to non-Mexican American students (*n* = 154) also indicated misfit and inadequate configural measurement invariance. Together, these findings underscore an emergent need to closely evaluate use of the MACVS beyond intended samples and acknowledge context-dependent influences on cultural values for Mexican American college students.

## Introduction

As of 2024, Latinx Americans constitute 20% of the United States (U.S.) population (68 million people) and 51% of the population growth, with Mexican Americans making up the largest proportion of this group (57%) [1]. This influx accompanies a large representation of Latinx Americans within higher education, with 46% having attended at least some college [1]. Across Latinx subgroups in the U.S., there is evidence that cultural values have important protective effects on mental and physical health [2]. Moreover, leveraging cultural factors in mental and physical health intervention and prevention efforts is a high priority in minority health and mitigating health disparities [3]. Although Latinx Americans experience significant acculturative, mental, and biological risk factors, they may do so at rates lower than what would be expected based on their exposure to adversities—a phenomenon known as the Hispanic Health Paradox [2,4–7]. Given that more recent research has called this paradox into question based on very high rates of post-traumatic stress among recent immigrants [8], and that any Latinx health advantage is not perfectly understood, several scholars cite Latinx cultural values–such as cultural supports, feelings of belonging, and protections–as possible explanations [7,9].

Against this background, the current study aimed to evaluate one such instrument, the Mexican American Cultural Values Scale (MACVS) [10], for use among Mexican American college students. Surprisingly, the MACVS has not been psychometrically evaluated specifically for Mexican American college samples. Yet, the MACVS has been implemented to account for cultural values across Mexican American subpopulations, as evidenced by a wealth of studies across adult [11] and adolescent samples [12–16]. Moreover, the MACVS is among the most cited cultural values measurement, with over 400 direct citations as of 2021 [17]. In light of a growing Mexican American population with experiences that may differ significantly from those of immigrant populations, a more thorough psychometric evaluation is required when utilizing the MACVS for college student populations. Indeed, calls have been made for more rigorous psychometric testing of acculturation and cultural value scales [18]. Given the rise of Mexican American Latinx populations in the U.S. and variation among Mexican American experiences there is a critical need for instruments that can assess cultural values across such groups.

### Initial development and psychometric properties of the MACVS

Development of the MACVS was initially reported within Knight and colleagues’ focus group examination of bicultural cultural values across two samples comprising adolescent Mexican Americans, their mothers, and their fathers [10]. Originally composed of a 63-item scale, the MACVS was trimmed to 50 items, and now addresses nine themes generally capturing Traditional Values and Mainstream Values: *Familism-Support, Familism-Obligation, Familism-Referent, Respect, Religion, Traditional Gender Roles, Material Success, Independence & Self-Reliance,* and *Competition & Personal Achievement*. *Familism-Support* describes values prioritizing familial prosperity and unity, and is linked to resiliency against prejudice and discrimination [19,20]. *Familism-Obligations* entails a sense of responsibility to support family members. *Familism-Referent* describes advice-seeking from other family members, typically elders. *Respect* reflects adherence to rules set by family members. *Religion* reflects endorsement of spiritual beliefs.*Traditional Family Roles* describes gender role expectations prescribed for male and female family members. *Material Success* describes prioritizing monetary gains over other activities, such as having higher economic expectations for oneself. *Independence & Self-Reliance* reflects self-sufficiency. Finally, *Competition & Personal Achievement* demonstrates the separation of oneself from others through personal advancement [19].

Tests of model fit published regarding the MACVS indicated that these factors fit best when comprised of two higher-order factors: Traditional Beliefs and Mainstream Values [19]. The first factor, Traditional Beliefs, captures six first-order factors reflecting Mexican and Mexican American values: *Familism-Support, Familism-Obligations, Familism-Referent, Respect,* *Religion,* and *Traditional Gender Roles*. The second factor, Mainstream Values, describes contemporary American values and comprises three factors: *Material Success, Independence & Self-Reliance,* and *Competition & Personal Achievement*. Previous tests of model fit demonstrated that allowing the two higher-order factors to correlate yielded better fit indices (within the recommended cut-off for two of three fit indices) than when uncorrelated or independent. Thus, the final model captures a dual cultural adaptation framework reflecting the simultaneous attainment of American values and retention of Traditional values [19].

Cultural values measured using the MACVS have been useful for examining how Mexican Americans experience and interact with their environment. For example, *Familism-Support* is linked to resiliency against prejudice and discrimination [19,20]. *Familism-Support,* in one study, buffered the association between perceived discrimination and suicide-related thoughts [21]. *Familism-Referent* endorsement was associated with reduced internalizing behavior [22,23] and reduced substance abuse and deviancy [24]. More broadly, Gonzalez and colleagues demonstrated that endorsing Traditional Values in adolescent Mexican Americans led to increased academic engagement and fewer externalizing problems [25]. In fact, cultural values mediated the relationship between immigrant status and these outcomes, suggesting that endorsement serves a protective role [21,25].

### The need for measurement invariance testing for Mexican American college students

Despite the vast utility of the MACVS across diverse Latinx subgroups and outcomes, our current understanding of its psychometric properties for Mexican American college students is limited. Scholars argue that Mexican American populations exhibit heterogeneous value orientations due to heterogenous acculturative experiences, which may play a role in health behavior outcomes [26]. Moreover, a growing body of literature has called for the psychometric evaluation of assessments within Latinx groups with respect to early childhood language, heritage-culture retention, and ethnic pride [27–29]. Psychometric evaluation of the MACVS is important, as the scale continues to be utilized across many clinical domains, which, in turn, has profound implications on our understanding of health within these populations, such as influencing parental beliefs in the treatment of attention-deficit disorder, suicide behavior, among others [21,23,30].

### Other considerations for non-Mexican American college samples

Additionally, Mexican Americans constitute only a portion of descendants from Latin American countries living in the U.S and attending higher education. Other countries (e.g., Puerto Rico, Guatemala, and El Salvador) represent growing segments of the Latinx population in the U.S., with critical cultural distinctions in language, acculturation experiences, and attitudes toward healthcare [31,32]. Further, studies that do account for nationality have found heterogeneity in the health and clinical outcomes of Latinx Americans [33–36]. Recognizing heterogeneity within Latinx subgroups calls into question the internal consistency of the MACVS and whether it is a comparable measure of cultural value among college-attending non-Mexican American samples, in addition to college-attending Mexican American samples.

On the one hand, values identified by the MACVS do appear in many Latinx cultural groups [37,38], and the protective effects of these cultural values have been observed across samples of varied national origins [39,40], suggesting it holds promise as a valuable instrument across Latinx subgroups. While this research implies that the MACVS may be applied to a broader Latinx population, psychological testing is necessary to establish researcher and clinician confidence in the derived conclusions.

To our knowledge, the MACVS has not been psychometrically tested or reevaluated since its conceptualization for college students. The MACVS was initially developed to compare cultural values among adult and adolescent Mexican American samples, specifically adolescent Mexican Americans and their parents [10], but did not account for education level and college attendance. Therefore, establishing the cross-national measurement invariance of the MACVS is key to advancing its use as a general measure of Latinx cultural values beyond adolescent and caregiver Mexican American populations.

### The current study

The overarching goal of the current study is to demonstrate the psychometric utility of the MACVS among Mexican American college students. This objective was completed through two steps. First, we evaluated the discriminant validity between the MACVS and two constructs: Perceived Ethnic Discrimination (PEDQ) [41] and the Short Acculturation Scale (SAS) [42] among a sample of Mexican American college students. The PEDQ and SAS have demonstrated success in understanding downstream Latinx health disparities [42,43]. For instance, the PEDQ is useful for examining links between perceiving discrimination and alcohol consumption and depressive symptoms [43]. Of note, the PEDQ has been used alongside the MACVS to understand the protective effects of cultural values, and, in particular, *Familism-Support* has been reported to buffer against depressive symptoms and suicide behavior among Latinx populations perceiving discrimination [21].

#### Hypothesis 1.

We anticipate finding negative correlations between Traditional Values and the PEDQ, as well as between Traditional Values and SAS. Additionally, we expect to find positive correlations between the Mainstream Values and SAS. Taken together, these findings would provide convergent validity and support for the utility of the MACVS beyond adolescent Mexican Americans and caregivers to Mexican American college students. Additionally, we explored the association between PEDQ and Mainstream Values.

#### Hypothesis 2.

We then sought to examine the psychometric properties of the MACVS among Mexican American college samples, specifically, its internal consistency, reliability, factor structure, and measurement invariance using the correlated higher-order model.

Based on prior literature [10] and the intended use of the MACVS among Latinx college-attending populations, we expected an adequate fit for the correlated higher-order model.

#### Exploratory analysis.

Following the primary psychometric validation in the Mexican American college sample, we then conducted a secondary set of analyses in a comparison sample of non-Mexican American college students. This exploratory analysis was performed out of acknowledgement of emerging literature that broadly applies the MACVS to non-Mexican American populations. We examined the factor structure of the MACVS and tested for measurement invariance across the two subgroups. Similar to the Mexican American college sample, we began by evaluating the reliability and correlations of the nine first-order factors within each subgroup and in the combined sample.

Finally, to assess the potential broader applicability of the MACVS, we conducted sequential tests of measurement invariance between the college-attending Mexican American and non-Mexican American groups.

## Materials and methods

### Ethics statement

The data collect was approved by the Sam Houston State University – Institutional Review Board (protocol # 2017-04-34345). Participants provided written informed consent prior to engaging with the online survey.

### Participants and procedure

Data were collected from a larger study examining mental health in university students whose family history emigrated from Spanish-speaking countries [21,44,45]. The complete sample included 3425 undergraduate student participants enrolled in seven universities within Texas, U.S.. All participants were at least 18 years old at the time of data collection. Of these, 585 were included if they had answered the demographic question, “What is your country of origin?” Ninety responses were then excluded due to missing data using list-wise deletion. Thus, the total sample for data analysis consisted of 495 responses. All measures were administered in English.

#### Mexican American college sample.

A total of 341 participants identified their country of origin as Mexico, representing the Mexican American college sample. Most were female (78.9%) and aged 18–55 years old (*M* = 22.13, *SD* = 5.66). Additionally, the majority (55.6%) identified as U.S. citizens and reported English as their native language (58.8%). Of those who were not born in the U.S., mean age of emigration was 6.27 years (*SD* = 5.12).

#### Non-Mexican American college sample.

A total of 154 participants identified as Hispanic or Latinx but were not of Mexican origin, representing the non-Mexican American college sample. Participants indicated their country of origin as one of several Latin American countries: 5.7% El Salvador, 5.7% Puerto Rico, 4.8% Colombia, 3.2% Venezuela, and 1.6% Honduras. Participant ages ranged between 18 and 55 (*M* = 22.27, *SD* = 6.11). The majority were female (84.3%), identified as U.S. citizens (65.4%), and English as their native language (53.1%). The mean age of emigration for non-Mexican American participants who were not born in the U.S. was 9.93 years (*SD* = 7.50).

### Measure

#### Demographic form.

As part of the larger study, the investigators also collected relevant demographic information, such as ethnicity, the current year in university, and parent education levels. Additionally, the decision to combine of non-Mexican countries of Hispanic or Latin descent into a singular “non-Mexican” was made out of statistical necessity to ensure statistical rigor for the subsequent psychometric tests. Combining this group does not indicate or suggest that these countries share universal cultural values and the results should be interpreted with caution. All measures and materials were approved by university Institutional Review Board prior to study initiation and participants provided informed consent before completing the survey.

#### Mexican American Cultural Values Scale.

The 50-item Mexican American Cultural Values Scale (MACVS) [10] comprises nine factors examining differential Latinx cultural expectations. Six factors specified Mexican or Mexican American values: *Respect* (6 items; e.g., “No matter what, children should always treat their parents with respect.”), *Religion* (7 items; e.g., “One’s belief in God gives inner strength and meaning to life.”), *Traditional Family Roles* (6 items; e.g., “Men should earn most of the money for the family so women can stay home and take care of the children and the home.”), *Familism-Obligations* (5 items; e.g., “If a relative is having a hard time financially, one should help them out if possible.”), *Familism-Support* (6 items; e.g., “It is important to have close relationships with aunts/uncles, grandparents, and cousins.”), and *Familism-Referent* (5 items; e.g., “When it comes to important decisions, the family should ask for advice from close relatives."). The six subscale scores achieved good internal consistency (*Respect* α = .87, *Religion* α = .97, *Traditional Family Roles* α = .76, *Familism-Obligations* α = .72, *Familism-Support* α = .86, and *Familism-Referent* α = .86).

Mainstream Values are attributed to American mainstream culture. Mainstream Values are composed of three subscales, including *Independence & Self-Reliance* (α = .64; e.g., “The most important thing parents can teach their children is to be independent of others.”), and *Competition & Personal Achievement* (α = .67; e.g., “One must be ready to compete with others to get ahead.”), and *Material Success* (α = .85; e.g., “Children should be taught that it is important to have a lot of money.”). *Material Success* and *Independence & Self-Reliance* comprised five items, while *Competition & Personal Achievement* comprised four items. The scale is scored using a 5-point Likert scale ranging from 1 (not at all) to 5 (completely), with higher scores reflecting a higher presence of respective cultural values. Cronbach’s alpha for each subscale across subgroups is provided in Table 1.

**Table 1:** Cronbach’s Alpha values and composite reliability for MACVS subscales and factors by sample.

Factor	Mexican Sample		non-Mexican Sample		Total Sample	
	N = 341		N = 154		N = 495	
	α	ρc	α	ρc	α	ρc
**Traditional Values**	0.95	–	0.94	–	0.94	–
**Familism-Support**	0.86	0.86	0.85	0.85	0.85	0.86
**Familism-Obligations**	0.72	0.72	0.72	0.74	0.72	0.72
**Familism-Referent**	0.79	0.8	0.79	0.79	0.79	0.8
**Respect**	0.87	0.87	0.87	0.87	0.87	0.87
**Religion**	0.97	0.97	0.97	0.97	0.97	0.97
**Traditional Gender Roles**	0.78	0.79	0.68	0.69	0.76	0.77
**Mainstream Values**	0.80	–	0.85	–	0.82	–
**Material Success**	0.84	0.84	0.86	0.87	0.85	0.85
**Independence & Self-Reliance**	0.61	0.63	0.66	0.69	0.63	0.65

#### Perceived ethnic discrimination questionnaire.

The 18-item Perceived Ethnic Discrimination Questionnaire (PEDQ) [46] is composed of five factors evaluating perceived racism and ethnic discrimination: lifetime exposure, exclusion/rejection, stigmatization/devaluation, discrimination, and threats/aggression. Response values were 1 = Never Happened, 3 = Sometimes, and 5 = Happened Very Often, with higher averages reflecting a higher perception of racism. Cronbach’s alpha for the overall PEDQ were α = .92 for the total sample, α = .93 for the Mexican American college sample, and α .91 for the non-Mexican American college sample.

#### Short acculturation scale.

The 12-item Short Acculturation Scale (SAS) [42] evaluates language fluency (i.e., 1 = Only English, 2 = English better than Spanish, 3 = Both Equally, 4 = Spanish better than English, and 5 = Spanish Only). Cronbach’s alpha for the overall SAS of the total college sample was α = .84, α = .83 for the Mexican American sample, and α =  .84 for the non-Mexican American college sample.

### Data analysis overview

#### Primary analysis.

Cases with missing data were filtered and removed list-wise prior to data analysis. Preliminary data were assessed for normality before conducting internal consistency and Confirmatory Factor Analysis (CFA). Skewness and kurtosis were below the suggested cutoff of +/−2 for skewness and +/−7 for kurtosis, suggesting univariate normality [47]. Internal consistency of the MACVS across both samples included reliability and factor correlation tests. Convergent validity was also explored utilizing the PEDQ and the SAS scales to examine perceived discrimination and acculturation, respectively, in the subgroups (Hypothesis 1).

CFA and measurement invariance testing were performed utilizing a maximum likelihood robust to non-normality estimation in which configural, metric, and scalar invariance were examined. CFAs for each model were tested sequentially, beginning with the nine independent-factor model and the nine correlated-factor model, and then measuring the invariance of the nine correlated-factor model. Tests of the independent higher-order factor model and the recommended correlated higher-order factor model [10] were then examined (Hypothesis 2).

Model fit statistics were as follows: chi-square goodness-of-fit test (χ^2^), Root Mean Square Error of Approximation (RMSEA), Comparative Fit Index (CFI), Tucker Lewis Index (TLI), and Standardized Root Mean Square Residual (SRMR). Specifically, the required cutoff criteria for acceptable model fit were CFI ≥ 0.90, TLI ≥ 0.90, RMSEA ≤ 0.08, and SRMR ≤ .08. Excellent model fit required CFI ≥ 0.95, TLI ≥ 0.95, and RMSEA ≤ 0.06 [48].

#### Exploratory analysis.

Measurement invariance across the Mexican respondents was then examined through a hierarchical, step-wise approach fitting more restrictive model criteria [49]. This bottom-up sequence, in which additional constraints are added, is recommended mainly for invariance model testing when higher-order factor loadings are present [49]. Configural invariance was used to examine the baseline model which tests whether the MACVS and subscales were invariant across both groups. Next, metric (weak factorial) invariance was tested, whereby factor variances were allowed to remain free, but factor loadings were held invariant across groups. Establishing metric invariance suggests that covariances between first-order factors and loadings of the higher-order factors are compared across groups. Finally, scalar invariance tests (strong factorial) provide additional constraints in which indicator intercepts and factor loadings are held invariant across groups. Rudnev and colleagues posited that measurement invariance of the first-order models must be established before invariance tests of the higher-order factor models [49]. This series of steps was then repeated for the non-Mexican American respondents. Given the sensitive nature of the sample, the data and materials may be requested through the corresponding author. The analysis code for this study is also available upon request through the first author.

## Results

### Preliminary analyses and descriptive statistics of the MACVS

Table 2 shows the mean, standard deviation, skewness, kurtosis, and group comparisons for the factor (i.e., Mainstream Values and Traditional Values) for each subscale across the Mexican American as well as the non-Mexican American and total college samples for supplemental analyses. See Table 2 for subscale correlations. Sample item means, standard deviations, and item-total correlations (ITCs) can be found in Table 2.

**Table 2:** Means, standard deviations, skewness, and kurtosis of MACVS subscales across Mexican, non-Mexican, and total sample.

	Mexican American *N* = 341				non-Mexican American *N* = 154				Total sample *N* = 495			
Factor	*M*	*SD*	g1	g2	*M*	*SD*	g1	g2	*M*	*SD*	g1	g2
Mexican American Values Subscales												
Familism-Support	24.42	4.49	−0.81	0.43	24.22	4.34	−0.67	0.02	24.36	4.44	−0.77	0.32
Familism-Obligations	19.03	3.58	−0.43	−0.20	18.64	3.41	−0.26	−0.07	18.91	3.53	−0.37	−0.17
Familism-Referent	17.11	4.14	−0.33	−0.38	16.62	3.97	−0.20	−0.21	16.96	4.09	−0.28	−0.33
Respect	29.31	6.14	−0.45	−0.46	28.38	6.33	−0.57	0.34	29.02	6.21	−0.50	−0.15
Religion	23.25	9.53	−0.39	−1.23	23.07	9.82	−0.33	−1.36	23.19	9.61	−0.37	−1.26
Traditional Gender Roles	9.47	4.04	1.14	1.17	9.03	3.49	1.00	0.44	9.33	3.88	1.13	1.15
Overall Mexican American Values	122.59	24.55	−0.33	−0.48	119.95	23.51	−0.30	−0.12	121.77	24.24	−0.32	−0.37
Mainstream Values Subscales												
Material Success	9.46	4.12	1.03	0.63	9.56	4.15	0.87	0.34	9.49	4.12	0.98	0.55
Independence & Self-Reliance	18.84	3.22	−0.15	−0.53	18.60	3.14	−0.12	−0.38	18.76	3.19	−0.14	−0.48
Competition & Personal Achievement	12.40	3.11	−0.10	0.10	12.04	2.94	−0.31	−0.04	12.28	3.06	−0.15	0.10
Overall Mainstream Values	40.69	7.83	0.48	0.74	40.21	7.94	0.34	0.00	40.54	7.86	0.44	0.52

*M* = Mean; *SD* = Standard Deviation, g1 = Skewness, g2 = Kurtosis.

### Internal consistency

Almost all inter-subscale correlations were moderate, and the composite Traditional Values and Mainstream Values were positively correlated (see Table 3). Specifically, within the Mexican American college sample, the Mainstream Value subscales *Independence & Self-Reliance, Material Success,* and *Competition & Personal Achievement* were positively correlated with one another. All Mainstream Value subscales also held positive correlations with many Traditional Value subscales, except with *Religion*, which was not correlated with any Mainstream Value subscale. Specifically, *Material Success* held positive correlations with *Familism-Referent, Familism-Respect,* and *Traditional Gender Roles,* but not *Familism-Support, Familism-Obligations* or *Religion.* The subscale *Independence* was positively correlated with *Familism-Support, Familism-Obligation, Familism-Referent, Respect,* but not *Traditional Gender* *Roles* or *Religion.* The third Mainstream Value subscale, *Competition & Personal Achievement,* was positively correlated with all subscales, except for *Religion*. MACVS item means, standard deviations, and total correlations are presented in Table 4.

**Table 3:** Factor correlations.

	1	2	3	4	5	6	7	8	9	10	11	12	13
**1. Traditional Values**	--	.736^***^	.769^***^	.809^***^	.771^***^	.835^***^	.470^***^	.132	−.006	.089	.207^**^	−.166 ^*^	.073
**2. Familism-Support**	.779^***^	--	.738^***^	.650^***^	.345^***^	.633 ^***^	.114	−.059	−.242^**^	.081	.030^***^	−.325^***^	.031
**3. Familism-Obligations**	.786^***^	.771^***^	--	.704^***^	.372^***^	.654^***^	.264^***^	.097	−.084	.088	.210 ^***^	−.180^**^	.037
**4. Familism-Referent**	.853^***^	.708^***^	.740^***^	--	.412^***^	.699^***^	.393^***^	.202^***^	.092	.050	.291^***^	−.064	.048
**5. Religion**	.797^***^	.487^***^	.428^***^	.525^***^	--	.474^***^	.304^***^	.011	−.025	−.005	.053	−.031	.096
**6. Respect**	.853^***^	.675^***^	.705^***^	.759^***^	.508^***^	--	.267^***^	.120	−.079	.153	.203 *	−.200^*^	−.013
**7. Traditional Gender Roles**	.447^***^	.069	.210^***^	.353^***^	.256^***^	.318^***^	--	.393^***^	.471^***^	.053	.307^***^	−.015	.194*
**8. Mainstream Values**	.297^***^	.140**	.220^***^	.344^***^	.058	.322^***^	.462^***^	--	.822^***^	.680^***^	.850^***^	.138	.058
**9. Material Success**	.169^**^	−.073	.039	.205^***^	.026	.155^**^	.570^***^	.785^***^	--	.274^***^	.590^***^	.178^*^	.104
**10. Independence**	.214^***^	.256^***^	.250^***^	.216^***^	.019	.284^***^	.039^***^	.634^***^	.161^**^	--	.445^***^	.074	−.095
**11. Competition & Self-Reliance**	.301^***^	.188**	.284^***^	.380^***^	.080	.316^***^	.393^***^	.829^***^	.524^***^	.375^***^	--	.087	.092
**12. PEDQ**	−.069	−.103^*^	−.031	−.027	−.103^*^	−.055	.085	.134^*^	.132*	.075	.090	--	.030
**13. SAS**	−.044	−.058	−.071	.015	−.080	−.020	.027	.091	.109^*^	.042	.041	−.001	--

Below diagonal is Mexican Sample, above is non-Mexican sample.

**Table 4:** Item-total correlations.

	Mexican			non-Mexican			Total Sample		
Item	*M*	*SD*	Item-Total Correlation	*M*	*SD*	Item-Total Correlation	*M*	*SD*	Item-Total Correlation
**1**	3.54	1.41	.52	3.55	1.41	.55	3.54	1.41	.53
**2**	3.99	1.03	.62	3.90	.97	.50	3.96	1.01	.59
**3**	3.60	1.22	.58	3.56	1.15	.52	3.59	1.20	.57
**4**	2.76	1.13	.55	2.73	1.07	.49	2.75	1.11	.53
**5**	4.21	1.07	.58	4.07	1.19	.41	4.16	1.11	.53
**6**	2.24	1.10	.31	2.25	1.07	.22	2.24	1.09	.29
**7**	4.25	.92	.36	4.14	.86	.13	4.22	.90	.30
**8**	2.94	1.55	.60	2.94	1.66	.56	2.94	1.58	.59
**9**	4.05	1.04	.56	3.99	1.02	.38	4.03	1.04	.51
**10**	3.72	1.19	.60	3.62	1.18	.53	3.69	1.19	.58
**11**	3.69	.95	.45	3.60	.91	.39	3.66	.94	.43
**12**	3.19	1.15	.53	3.18	1.07	.42	3.19	1.12	.50
**13**	1.67	1.11	.35	1.57	.98	.32	1.64	1.07	.34
**14**	3.20	1.24	.28	3.21	1.20	.21	3.20	1.23	.26
**15**	2.39	1.12	.54	2.42	1.12	.50	2.40	1.12	.53
**16**	1.97	1.12	.21	2.00	1.07	.10	1.98	1.10	.18
**17**	3.98	1.03	.26	3.97	.93	.29	3.98	1.00	.27
**18**	3.26	1.43	.68	3.24	1.51	.65	3.25	1.46	.67
**19**	2.26	1.13	.35	2.16	1.14	.28	2.23	1.13	.33
**20**	4.30	.87	.54	4.19	.96	.45	4.26	.90	.51
**21**	3.72	1.06	.48	3.67	1.02	.47	3.71	1.05	.48
**22**	4.31	.86	.41	4.23	.87	.46	4.29	.86	.42
**23**	3.02	1.24	.45	2.88	1.13	.34	2.98	1.21	.42
**24**	2.04	1.09	.32	2.10	1.16	.19	2.06	1.11	.27
**25**	3.70	1.15	.71	3.35	1.22	.65	3.59	1.18	.69
**26**	4.27	.85	.08	4.31	.72	.11	4.28	.81	.08
**27**	3.56	1.49	.61	3.52	1.47	.60	3.55	1.49	.61
**28**	3.75	1.06	.59	3.86	.98	.47	3.79	1.04	.55
**29**	3.94	1.02	.54	3.80	.95	.53	3.89	1.00	.54
**30**	3.66	1.18	.73	3.55	1.10	.63	3.63	1.16	.70
**31**	3.25	1.11	.61	3.15	1.13	.55	3.22	1.11	.59
**32**	1.52	1.02	.34	1.35	.83	.14	1.47	.96	.29
**33**	3.41	1.16	.25	3.21	1.11	.31	3.35	1.15	.27
**34**	1.59	.98	.25	1.62	.94	.28	1.60	.97	.26
**35**	2.63	1.29	.04	2.53	1.29	.00	2.60	1.29	.03
**36**	3.57	1.51	.62	3.57	1.55	.65	3.57	1.52	.62
**37**	4.07	1.00	.49	3.99	1.00	.52	4.05	1.00	.50
**38**	4.09	.93	.41	4.01	.87	.38	4.06	.91	.40
**39**	3.84	1.01	.55	3.68	.96	.58	3.79	1.00	.56
**40**	3.48	1.12	.67	3.36	1.10	.63	3.45	1.12	.66
**41**	2.70	1.20	.37	2.79	1.14	.38	2.73	1.18	.37
**42**	2.09	1.18	.41	1.90	1.14	.32	2.03	1.17	.38
**43**	1.61	.98	.24	1.59	.90	.11	1.61	.96	.21
**44**	3.70	1.01	.28	3.64	.92	.28	3.68	.98	.28
**45**	3.23	1.51	.66	3.14	1.57	.65	3.20	1.53	.66
**46**	4.27	.85	.40	4.29	.82	.47	4.27	.84	.42
**47**	3.65	1.15	.64	3.47	1.20	.65	3.60	1.17	.64
**48**	3.16	1.48	.66	3.11	1.49	.63	3.14	1.48	.65
**49**	4.24	.87	.51	4.18	.93	.64	4.22	.89	.55
**50**	1.92	1.09	.31	2.05	1.13	.52	1.96	1.11	.37

*M* = Mean; *SD* = Standard Deviation.

Interpretation of Cronbach’s alpha values is based on George and Mallery’s [50] guidelines (i.e., > .9 – Excellent, > .8 – Good, > .7 – Acceptable, > .6 – Questionable, > .5 – Poor, and <  .5 – Unacceptable). In the Mexican American college sample, values ranged between acceptable and excellent for all Mexican American Values subscales and *Material Success* from the Mainstream Value subscales. For the Mainstream Value subscales *Independence & Self-Reliance* and *Competition & Personal Achievement,* Cronbach’s alpha values were in the questionable range (see Table 1).

In addition to Cronbach’s alpha, composite reliability (ρc) was computed using CFA standardized loadings to provide a model-based estimate of internal consistency that is more appropriate for latent-variable models and relatively short subscales. Composite reliability estimates for the total, Mexican American, and non-Mexican American college samples are presented in Table 1. Across all three samples, composite reliability values were generally acceptable. *Religion* demonstrated consistently strong reliability, whereas *Independence & Self-Reliance* showed comparatively lower reliability across samples. Importantly, reliability estimates were highly similar across the three samples.

### Validity

Lastly, convergent validity was established. According to Hypothesis 1, we expected a negative correlation between Traditional Values and the PEDQ among the Mexican American college-student sample. We also anticipated that the SAS would be negatively correlated with Traditional Values and positively correlated with the Mainstream Values subscales. First, the SAS and PEDQ were not significantly correlated with one another (*r* = −.08, *p* = .77). Contrary to expectations, there was a non-significant correlation between Traditional Values and the PEDQ (*r* = −.08, *p* = .32) and the SAS (*r* = −.05, *p* = .39). There were positive correlations between the composite Mainstream Values and PEDQ (*r* = .09, *p* = .08), but, consistent with Hypothesis 1, between Mainstream Values and the SAS (*r* = .10, *p* = .04). When examining correlations between the PEDQ, SAS, and MACVS subscales, the PEDQ was correlated negatively with *Familism-Support* (*p* = .05)*, Religion* (*p* = .05), and positively with *Material Success* (*p* = .009). The SAS was only correlated positively with *Material Success* (*p* = .04)*.* Taken together, Hypothesis 1 was partially supported.

### MACVS of independent and correlated first-order CFA

Confirmatory factor analysis of the nine independent factor model and the nine correlated factor model yielded non-positive definite terms within the error covariance matrix. Consistent with recommendations from Rudnev et al. (2018), we fixed one-factor loading within each of the nine first-ordered factors to 1: item 1 for *Religion*, item 2 for *Familism-Support*, Item 3 for *Familism-Obligations*, item 4 for *Familism-Referent*, item 5 for *Respect*, item 6 for *Material Success,* item 7 for *Independence and Self Reliance*, item 13 for *Traditional Gender Roles*, and item 14 for *Competition & Personal Achievement*.

However, both models continued to produce non-positive definite terms within the error covariance matrix and yielded a less than acceptable model fit (see Table 5), thereby qualifying the exploration of the subscales. Specifically, given that two Mainstream Values subscales (*Independence & Self-Reliance and Material Success* & *Personal Achievemen*t) held low correlations with all other factors and held questionable reliability, a CFA was performed on the Mainstream Values within the Mexican American sample. This CFA indicated an excellent model fit for the Mexican American sample: CFI = 0.99, TLI = 0.97, RMSEA = 0.06, SRMR = 0.02. The CFAs for the Traditional Values were also excellent. Despite questionable model fit following the independent and correlated CFA models, and following recommendations of Knight and colleagues in favor of higher-order models, testing of these models proceeded [10].

**Table 5:** Comparisons of model fit across the total, Mexican American, and non-Mexican samples.

Latent Model	*X* ^*2*^	CFI	TLI	RMSEA	SRMR
**Total Sample**	–	–	–	–	–
**9 Independent Factors**	(1148) = 3307.85*	0.86	.85	0.08*	0.19
**9 Correlated Factors**	(1148) = 3307.85*	0.85	.84	0.06*	0.20
**2 Independent Higher Order Factors**	(1165) = 3552.28*	0.83	.83	0.06*	0.10
**2 Correlated Higher Order Factors**	(1166) = 3563.41*	0.83	.82	0.06*	0.10
**Mexican Sample**	–	–	–	–	–
**9 Independent Factors**	–	–	–	–	–
**9 Correlated Factors**	–	–	–	–	–
**2 Independent Higher Order Factors**	(1176) = 3951.72*	0.73	.71	0.08*	0.18
**2 Correlated Higher Order Factors**	(1176) = 3951.72*	0.73	.71	0.08*	0.18
**Non-Mexican Sample**	–	–	–	–	–
**9 Independent Factors**	(1148) = 2252.41*	0.77	.75	0.08*	0.22
**9 Correlated Factors**	(1148) = 2252.41*	0.77	.75	0.08*	0.22
**2 Independent Higher Order Factors**	(1176) = 2753.53*	0.67	.66	0.09*	0.18
**2 Correlated Higher Order Factors**	(1176) = 2753.53*	0.67	.65	0.09*	0.18

*X²* = Chi square test statistic; CFI = Comparative Fit Index; TLI = Tucker Lewis Index; RMSEA = Root Mean Square Error of Approximation; SRMR = Standardized Root Mean Square Residual; Model fit for the 9 Independent Factor Model and the 9 Correlated Factor Model yielded not positive definite results. p < .001^*^.

### CFA of the independent and correlated two-factor higher-order models

The independent two-factor higher-order model for the Mexican American sample with fixed factor loadings converged normally, but resulted in less than acceptable fit. Lastly, the final, recommended model which comprised of two correlated higher-order factors, Traditional Beliefs and Mainstream Values [10], converged normally but produced less than acceptable fit indices (see Table 5).

In summary, CFAs were performed first on the lower, first-ordered models before proceeding to the the higher-order models, including independent and correlated two-factor higher-order models. Both first-order models, which tested the nine-factor model in which factors were uncorrelated and the nine-factor correlated first-factor model, failed to converge normally and produced non-positive definite indices. Subsequent analyses of two CFAs, one for Mainstream Values and one for Traditional Values both had excellent fit. In both cases of the two-factor higher-ordered independent and correlated models, CFAs converged normally but produced less than acceptable fit. Areas of concern again emerged within Mainstream Values.

### Exploratory analyses: Is it only for Mexican college students?

#### Testing of the MACVS internal consistency and reliability for non-Mexican American college students.

We then explored the relationship between the MACVS, the PEDQ, and the SAS to determine whether the MACVS operated similarly between Mexican college samples and non-Mexican American college samples. Thus, we explored correlations between the PEDQ and Mainstream Values subscales. Indeed, for the non-Mexican American sample, the PEDQ was moderately and negatively correlated with the composite Traditional Values score (*r* = .17), and subscales *Familism-Support* (*r* = −.33), *Familism-Obligations* (*r* = −.18), and *Respect* (*r* = −.20). Similar to the Mexican American sample, total PEDQ was positively correlated with *Material Success* (*r* = .18). The total SAS was slightly and positively correlated with the *Familism-Support* subscale (*r =* .09, *p* = .05) and *Traditional Roles* subscale (*r* = .19).

Regarding internal consistency, subscales for the non-Mexican American sample were largely the same as the Mexican American sample. Notably, Cronbach’s alphas for all Mainstream Values subscales were higher for the Non-Mexican sample (see Table 2) than the Mexican American sample. Moreover, only the Traditional Gender Roles value was significantly lower for the non-Mexican sample.

#### CFA of the independent and correlated two-factor higher-order models.

**Non-Mexican American**

CFAs of the first-order nine independent and correlated models were then tested for the non-Mexican American (*n* = 154) sample. The CFAs of the independent nine-factor model and the correlated nine-factor model within the non-Mexican American sample converged normally, albeit with less than adequate fit for both (see Table 5). The recommended model with fixed factor loadings was then tested within the non-Mexican American sample. Tests for the correlated two-factor higher-order factor model within the non-Mexican American sample (with fixed factor loadings to 1) converged normally but resulted in inadequate model fit (see Table 5). For comparison, tests of the two-factor higher-order independent model results are also provided.

#### MACVS invariance testing of the first-order nine-factor model.

To determine utility of the MACVS for Mexican and non-Mexican American college students, it was then necessary to examine its measurement invariance between the samples. This began with configural invariance (baseline model) testing of the first-order nine-factor correlated model. However, this produced non-positive error disturbance terms between the *Familism-Support* and the *Material Success* subscales for the non-Mexican American sample and provided inadequate fit to the data: CFI = 0.82, TLI = 0.81, RMSEA = 0.07, SRMR = 0.19. As the model provided inadequate fit across measurement indices, the same basic factor structure could not be applied similarly to each group’s data [51].

#### MACVS invariance testing of the two-factor higher-order models.

Reported previously, Knight et al. [10] recommended a two-factor higher-order model, acknowledging limitations of the lower-order, nine-factor correlated model. Invariance testing of the recommended model and modifications yielded a normal conversion; however, configural invariance was unsuccessful: CFI = 0.71, TLI = 0.70, RMSEA = 0.09, SRMR = 0.18, indicating non-equivalence between Mexican American and non-Mexican American college students with regard to psychometric utility of the MACVS. Further testing to determine metric and scalar invariance was therefore not completed.

To further clarify the nature of the observed non-equivalence, supplementary diagnostic analyses were conducted using the converged configural model to examine cross-group differences in standardized factor loadings. Absolute loading differences ranged from small to moderate (|Δ| < .15 for most items), with larger discrepancies concentrated within the *Independence & Self-Reliance* and *Traditional Gender Roles* subscales (largest |Δ| values = .25–.33; e.g., MACVS item 26, MACVS item 35, MACVS item 32). These findings suggest that measurement non-equivalence was localized to specific dimensions rather than reflective of global structural failure of the MACVS.

### Total sample exploratory factor analyses

Given persistent lack of acceptable fit across confirmatory models, an exploratory factor analysis (EFA) was conducted with the total sample of Latinx college students. Sampling adequacy was excellent (KMO = .93), with all item-level MSAs ≥ .77. Bartlett’s test of sphericity was significant, *χ²*(1225) = 14,960.90, *p* < .001, supporting factorability of the correlation matrix.

Parallel analysis indicated retention of six factors. Accordingly, a maximum likelihood EFA with oblimin rotation was estimated. The six-factor solution demonstrated good absolute fit, RMSEA = 0.05 (*90% CI* [.045,  .051]), RMSR = .03, TLI = .896, and accounted for 50% of total item variance. Primary standardized loadings ranged from  .35 to  .96 across factors. Salient loadings were defined as |λ| ≥ .40; secondary loadings ≥ .30 were considered indicative of cross-loading. The mean item complexity was 1.7, indicating nontrivial cross-loadings among several indicators. Factor correlations ranged from −.18 to  .63, supporting the use of oblique rotation and indicating related but distinguishable dimensions.

Inspection of the pattern matrix revealed both structural preservation and reorganization relative to the original model. *Religion* and *Traditional Gender Roles* emerged as psychometrically coherent factors, with strong primary loadings and minimal cross-loadings. *Religion* emerged as a highly coherent factor, with uniformly strong loadings (|λ|s ≥ .87) and minimal cross-loading. *Traditional Gender Roles* also formed a distinct factor; however, two *Independence & Self-Reliance* items loaded negatively on this dimension, yielding a bipolar structure characterized by traditionalism versus personal independence. In contrast, the three familism subdimensions (*Familism-Support, Familism-Obligations, and Familism-Referent*) did not emerge as separable constructs; their indicators converged onto a single familism factor. *Respect* items loaded jointly with *Familism–Referent* indicators, forming a broader relational-interdependence dimension. Additionally, the three Mainstream Values subscales (*Material Success, Independence & Self-Reliance, and Competition & Personal Achievement*) collapsed into a single achievement-oriented factor rather than distinct first-order domains. A sixth factor reflected a bipolar structure characterized by *Traditional Gender Role* endorsement inversely associated with *Independence & Self-Reliance* indicators. The mean item complexity (1.7) indicated nontrivial cross-loadings across domains.

## Discussion

The MACVS is one of the most cited scales used to evaluate Traditional and Mainstream Values among Mexican and Mexican Americans youths and families [10,17]. Yet, the MACVS continues to be utilized beyond Mexican and Mexican Americans youths and families, warranting inspection of its psychometric properties among other populations such as Mexican Americans and non-Mexican Americans college students. The presented results provide convergent validity tests, internal reliability, CFA, and measurement invariance for Mexican Americans college students. At the broadest level, our results indicate psychometric problems with the MACVS for utility among Mexican American college students. Surprisingly, there is evidence of low internal consistency and problems replicating previously published factor structures in the Mexican college students, as summarized below.

First, regarding convergent validity, we hypothesized negative correlations between the PEDQ, SAS, and Traditional Value and positive correlations with the Mainstream Values subscales, given the literature that demonstrates a negative correlation between *Familism-Support* and PEDQ [21]. As expected, the PEDQ was positively correlated with Mainstream Values, particularly *Material Success*, and negatively correlated with *Familism-Support* and *Religion*. We also anticipated that the SAS would be negatively correlated with the Traditional Values and positively correlated with the Mainstream Values subscales, which would exemplify decreased adherence to Traditional Values and simultaneous adoption of American cultural values. The SAS was only correlated positively with the *Material Success* subscale within Mainstream Values. These findings suggest that greater adoption of American cultural values might correspond with increased perceived discriminatory experiences. On the other hand, Traditional Value endorsement of *Familism-Support* and *Religion* might correspond with decreased perceived discriminatory experiences, similar to patterns identified within previous literature [21]. Although these findings appear to align partially with previous literature regarding the MACVS, these patterns might also explain how diverse experiences Mexican American students shape the adoption and acculturation of American culture while attending higher education.

Tests of internal consistency suggested excellent performance of the Traditional Values subscales. Several of the Mainstream values subscales, however, had questionable internal consistency. Moreover, CFAs indicated inadequate model fit across tests, including the recommended (correlated two-factor higher-order model) and alternative models (i.e., the independent two-factor higher-order model, the independent nine-factor first-order model, and the correlated nine-factor first-order). CFAs for the correlated higher-order latent factors and tests of the alternative models yielded similar issues; CFAs failed to converge for our first-order models within the Mexican American sample

### Comparisons with previous psychometric evaluations of the MACVS

Our results were surprising, considering that the MACVS has demonstrated promise for testing Mexican American cultural values and has been utilized to examine clinical outcomes across health, education, and other outcomes. The initial development of the MACVS indicated that a higher-order model with two correlated higher-order factors fit significantly better than an independent model regarding fit [10]. Our correlated two-factor higher-order models were similar in terms of CFI but significantly worse in terms of RMSEA and SRMR compared to those reported by Knight and colleagues.

Our results prompted closer inspection and interpretation of reliability estimates and CFAs for each subscale within the sample. First, reliability tests using Cronbach’s alphas suggested that Mainstream Values (*Material Success, Independence & Self-Reliance*, and *Competition & Personal Achievement*) fell within a questionable range. This is substantiated by the lack of the ITC Item 35 (Total Sample), “When there are problems in my life, a person can only count on him or herself,” and low ITCs for Item 26, “As children get older, their parents should allow them to make their own decisions.” While this might raise suspicions about internal reliability within our samples’ Mainstream Value endorsement, these low reliabilities are consistent with the results provided by Knight and colleagues, which reported lower Cronbach’s alphas across most subscales [10]. For example, Cronbach’s alphas for *Familism-Obligation*, *Familism-Referent*, *Independence & Self-Reliance,* and *Competition & Personal Achievement* subscales did not exceed .7 (*La Puerta Study*) [10]. Moreover, *Familism-Obligations* and *Independence & Self-Reliance* subscale reliabilities did not exceed .6 (*La Familia Study*) [10]. Despite this concern, while the composite reliabilities for Traditional and Mainstream Values exceeded .8 across adolescent, mother, and father Mexican origin subgroups, the Cronbach’s alphas within our Mexican American group subscales were higher. Our internal reliability estimates using Cronbach’s alpha, coupled with consistent composite reliability coefficients across the Mexican American, non-Mexican American, and total samples, suggest that the psychometric instability observed in subsequent confirmatory and multi-group analyses is unlikely to reflect substantial differences in internal consistency across groups. Therefore, internal reliability is unlikely to account for the negative variance estimates observed.

Nevertheless, several speculative explanations may account for the low internal reliabilities observed among the Mainstream Values subscales. First, it is possible that Latinx college students experience relative ambivalence toward constructs such as *Material Success* and *Competition & Personal Achievement*. This pattern may be salient among college-attending young adults who are aware of the financial and social costs associated with higher education attainment. Ambivalence toward *Material Success* and *Competition & Personal Achievement* may be particularly salient for college-attending Latina women, for which *Traditional Gender Roles* endorsement may directly conflict with career aspirations. At the same time, *Traditional Gender Roles* endorsement may discourage Latino young adult men from excelling in higher education due to expectant obligations to enter the workforce and provide financial resources for the family [52]. Alternatively, these patterns might extend beyond college students, potentially prompting a theoretical reconsideration of how American identity is conceptualized within this population. Although the latter possibility is not the intended focus of this paper, taken together, these internal reliability estimates suggest that additional psychometric work is needed to better understand the MACVS, particularly prior to extending its application to college student samples.

A third possible explanation concerns the use of subscale scores rather than the composite Mainstream Values score. Concerns regarding the utilization of the Mainstream Values subscales are consistent with arguments advanced by Knight and colleagues, who cautioned against reliance on the individual subscale scores and, instead, recommended use of the composite score [10]. Therefore, this finding is not surprising and, rather, reflects artifacts of Mainstream Values. Taken together, the utilization of the Mainstream Values subscales appeared to be a consistent and prevalent concern across psychometric evaluations of the MACVS stemming back to its initial conceptualization. Given the relative recency of these findings, future research should continue to examine endorsement of Mainstream Values relative to Traditional Values among Latinx individuals, regardless of college attendance. Doing so would help determine whether these patterns are contextual shifts specific to college students or indicative of a broader social movement characterized by increasing ambivalence toward Mainstream Values.

### MACVS: Is it for Mexican college students?

As previously noted, determining the psychometric utility and boundaries of the MACVS is necessary and was the original goal of this manuscript, as it has been used among samples beyond the populations within its initial conceptualization. Measurement invariance testing was used to determine such boundaries, for example, its utility among Mexican American and non-Mexican American college students. Scholars assert that the establishment of invariance of first-order models (i.e., the nine independent and nine correlated first-order models) is necessary before invariance testing of the two-latent factor model employed by Knight and colleagues [10,49]. After establishing the properties of the MACVS for Mexican American college sample, we attempted to confirm the factorial validity of first-order models with the total sample and for the non-Mexican American sample. Consistent with modification strategies employed by Knight and colleagues, constraining the model improved fit indices for both the Mexican American and non-Mexican American samples [10]. However, measurement invariance testing indicated an inadequate model fit. In turn, invariance testing for the first-order correlated model and higher-order correlated model was inadequate at the configural level, suggesting that the factor structure could not be applied across groups. Specifically, CFAs yielded an inadequate fit and produced negative error variances within the non-Mexican American sample.

A possible reason for the inadequate configural invariance might have been the diverging correlations between the Mainstream Values and Traditional Values subscales across subsamples. Whereas for Mexican American college students, Traditional Values were positively and significantly correlated with Mainstream Values, for non-Mexican American college students, the two were not significantly correlated. Other notable differences were centered on the subscale *Material Success*, for which there were non-positive covariances. Among Mexican American college students, *Material Success* was positively correlated with many Traditional Value subscales, including *Familism-Referent, Respect,* and *Traditional Gender Roles*. On the contrary, among non-Mexican American college students, while *Material Success* was correlated positively with *Traditional Gender Roles*, it was negatively correlated with *Familism-Support*. One possible explanation entails differential perceptions and acceptance of *Material Success* between samples. One might speculate that *Material Success* is an important component for those who endorse *Traditional Gender Roles* among Mexican American college students. On the contrary. In comparison, non-Mexican Americans may view these behaviors as unimportant or, in some cases, negative.

Despite failing to attain configural invariance, EFAs tested examining the total sample are encouraging, albeit point to considerable reorganization of the MACVS conceptualization among college student samples. Although conceptually distinct, *Respect* items loaded alongside *Familism-Referent* indicators, suggesting partial structural preservation but a notable shift in how these constructs may be conceptualized by the present sample. Specifically, *Respect*, characterized by deference to authority figures, obedience, and adherence to hierarchical family roles, and *Familism-Referent*, which emphasizes decision making and behavior guided by family reputation and internalized familial standards, may function as more integrated dimensions. This pattern suggests that behavioral deference and family referent identity processes may operate jointly rather than as clearly differentiated constructs in this context. Additionally, a sixth factor emerged that reflected a bipolar structure, defined by opposing loadings from *Traditional Gender Role* and *Independence & Self-Reliance* items. This pattern suggests a potential re-conceptualization of how these domains are organized within the present sample.

Lastly, correlations between subscales and measures of acculturation and Latinx experiences (SAS, PEDQ) point to important divergences between Mexican and non-Mexican American college students that must also be recognized. As previously noted, findings suggest that addressing the value adoption among subpopulations such as Latinx college students are shaped by both proximal (e.g., college-attendance, need for monetary resources) or distal (e.g., socio-political environments, geographical distance from countries of origin) is essential to interpretation of the MACVS.

### Implications and further directions

These findings have significant implications for using the MACVS in diverse cultural contexts and interpreting its results. Poor performance in terms of internal consistency and hypothesized factor structure in our samples—Mexican American and non-Mexican American alike—challenges previous conceptions regarding the relationship between Mainstream Values and Traditional Values as a fixture of the Latinx population at-large. When separated, both higher-order factors held excellent fit indices. Yet, when tested together, model fit indices remained inadequate across all tested models and, in some cases, failed to converge altogether. Moreover, inter-factor correlations suggest differences in acculturative experiences that generally shape the endorsement or adoption of Mainstream Values for Mexican Americans college students.

Such findings have several implications for non-Mexican American samples, for which Mainstream Value endorsement differed in its reliability and internal consistency. Other literature provides potential reasons for these differences. Sanchez and colleagues suggest that Puerto Ricans were more likely to engage in extramarital affairs, whereas Mexicans were more likely to view this behavior negatively [53]. These differences might have downstream consequences on behavioral health. Indeed, studies accounting for nationality have found significant heterogeneity in the clinical outcomes of Hispanic and Latinx Americans [33,35,36]. Cuban Americans, for example, had lower treatment retention among Latinx subgroups seeking addiction recovery [54] and were less acculturated as compared to Mexican Americans [55]. While preliminary, these findings underscore the need for further research on Mainstream Value endorsement, opening up new avenues for exploration and understanding.

These results also serve to clarify cultural value assessments and utility among Latinx samples in general. Latinx populations are not a monolith and measures like the MACVS may contribute to the essentialization of value systems ascribed to this community [56]. Members of the Latinx community vary in their endorsement of traditional cultural values, many of which are rooted in histories of colonist oppression (i.e., patriarchal norms) and perpetuate stereotypes [56]. It is possible that the poor model fit of the MACVS within the current sample may be, in part, explained by generational differences in critical consciousness that may contribute to the endorsement of values beyond those traditionally ascribed to their Latinx identity [56]. To address recent scrutiny regarding cultural values assessments and their utility in a diverse population, we recommend future research to identify contemporary cultural values within the Latinx community and update measures like the MACVS to better reflect possible changes across Latinx subpopulations.

### Limitations

Participants were recruited from Texas Hispanic-serving institutions. This population differs from the groups studied by Knight and colleagues, which comprised Mexican American young children ages 4–11 and their parents (ages of parents were not provided) [10]. While psychometric evaluation and assessment of the non-Mexican American college students was not the primary aim of these analyses, the lack of configural invariance allows researchers to speculate on the uniqueness of this population compared to other Mexican American adults and children. It is also necessary to acknowledge the nuanced socio-political environment of Texas in which cultural value inheritance, adoption, and rejection are intertwined. Texas is comprised of 11 million Latinx individuals, comprising 40.3% of the population [57]. However, the proportion of the Latinxpopulation substantially varies by proximity to the Texas-Mexico border, ranging between 30% in northern Texas, such as Plano (16%) and Arlington (31%) to > 80% among cities close Texas-Mexico border such as Laredo (96%), McAllen (90%), or El Paso (83%). Moreover, the largest share of the Latinx population are of Mexican descent, comprising approximately 86% of the Texas Latinx population. Within this context, endorsement of cultural values may be shaped by immigration and identity development within American culture, including proximity to extended family members residing in the U.S. and in countries of origin, immigration-related stressors (e.g., deportation concerns) that heighten perceptions of safety, and the unique stressors college studentsface while navigating Mexican and American cultural systems. Whether these findings reflect a diversification of generationally influenced cultural values specific to this socio-political and geographic context or are broadly applicable to Hispanic/Latinx college students remains an open question.

A second limitation regards the decision to combine non-Mexican Latinx descents into a single group, which may be seen as antithetical to contemporary arguments for Latinx research that acknowledges the richness and nuanced traditions and value systems between Latin American and Hispanic countries. This decision was made to ensure CFA models held ample statistical power within the non-Mexican American sample. Indeed, inadequate CFA fit suggest that the non-Mexican American sample should not be regarded as a singular entity, but rather, these countries hold variant value systems. Such findings reinforce the emergent need to further evaluate cultural values among these countries with sufficient sample sizes to ensure statistical power, whether using the MACVS or otherwise.

Third, the Mexican American sample was significantly larger than the non-Mexican American sample. These unbalanced invariance groups might contribute to factor analysis. Regardless, sample sizes offer one possible limitation concerning measurement invariance issues. Further, the sample size may not explain the inadequate fit reported among our Mexican American sample CFA and alternative models. CFA sample requirements continue to vary, with some scholars suggesting that, at minimum, the sample size should be at least greater than 100 (and upwards of 300–1000 for ‘excellent models [58,59]. Both sample sizes met minimum recommendations yet continued to perform inadequately, indicating model misfit. Nonetheless, these results indicate a need for increased psychometric evaluation of the MACVS and related cultural values assessments for Latinx populations.

Lastly, and equally important, is that the MACVS within the current sample was presented in English. Difficulty reading scale items for non-native English speakers or bilingual English and Spanish speakers can alter meaning and contribute to low reliabilities. However, this limitation appears to be minimal, as reliabilities were consistent within the higher-order factors and, in fact, performed better than other psychometric tests of the MACVS. Additionally, the majority of participants reported English as their native language across the Non-Mexican and Mexican American samples (58.8% and 53.1%, respectively). Nevertheless, future studies should continue to account for language fluency in administration and subsequent interpretation of results when utilizing the MACVS in English.

## Conclusion

An increasingly diverse Latinx American population introduces gaps within our understanding of Latinx cultural values. This research aimed to investigate whether the MACVS can be used to examine the values of Mexican college students. Issues arose when confirming model fit for the Mexican American college student sample, prompting inspection of the subscale CFAs, reliabilities, and ITCs. Exploratory tests of measurement invariance between Mexican and non-Mexican American college student samples further revealed divergences in Mainstream Value endorsement between these two subpopulations. Overall, our inspection of Knight and colleagues higher-order two-factor and alternative models suggests that Mainstream Vales may not fit previous conceptions of acculturation for college-attending Mexican American students [10].

## Supporting information

S1 File MACVSanonymized. (CSV)
