# Supplementary material for: Mexican American Cultural Values Scale: Considerations for college students
Source: PLoS One. 2026 Sep 1;21(9):e0354954. doi: 10.1371/journal.pone.0354954 (PMC13533370; doi:10.1371/journal.pone.0354954)
Supplement: Supplementary file 1 [file pone.0354954.s002.docx]

I. Overall Evaluation

This study presents a systematic, rigorous, and highly necessary examination of the psychometric properties of the Mexican American Cultural Values Scale (MACVS) within a college student population. Given the scale's widespread use in related research, contrasted with the scarce literature supporting its applicability to university student samples, this work fills a critical knowledge gap in the field. The authors have employed appropriate and advanced statistical methods, including Confirmatory Factor Analysis (CFA) and multi-group measurement invariance testing. The overall research design is logical, and its execution is rigorous.

The study's findings—namely, that the MACVS exhibits significant psychometric problems in both Mexican American and non-Mexican Latinx college student samples—carry important cautionary and practical value for the field. The manuscript is well-written, clearly structured, and presents a compelling argument.

Overall, this is a high-quality study with the potential for significant impact. I strongly recommend this manuscript for publication following appropriate revisions. My specific recommendations are intended to further enhance the rigor and depth of the paper's arguments.

Recommendation: Accept with Minor Revisions

II. Major Strengths

Significance and Originality: This study addresses a common yet often overlooked problem in psychological research: the uncritical use of measurement tools in untested populations. By focusing on the specific and important group of college students, the study demonstrates high practical relevance and originality.

Methodological Rigor: The authors have correctly selected and executed a series of complex statistical analyses. The transparent reporting of poor model fit and the handling of technical issues such as negative error variances demonstrate the authors' solid psychometric expertise. This honesty and rigor are commendable.

Impact of Findings: The study's conclusions will directly influence future researchers' choice of instruments for assessing cultural values in Latinx college students. This can prevent erroneous conclusions drawn from inappropriate tools and thereby directly contribute to improving the quality of research in this area.

Theoretical Contribution: The discussion section is outstanding. It not only analyzes the technical findings but also links them to broader theoretical issues, such as the heterogeneity of the Latinx population, the complexity of acculturation theory, and the rise of critical consciousness in younger generations. This greatly enhances the theoretical depth and heuristic value of the paper.

III. Specific Points for Revision

Below are several specific recommendations for revision that I believe will further strengthen the quality of the manuscript.

1. Regarding the Treatment and Discussion of the "Non-Mexican" Sample

Observed Issue: The manuscript combines participants from various national origins (e.g., Salvadoran, Puerto Rican, Colombian) into a single "non-Mexican" group. At a methodological level, this creates an internal tension with the paper's core argument that the Latinx population is not a monolith. The high level of heterogeneity within this composite group may itself be a significant cause of the poor model fit and the failure of the measurement invariance tests.

Suggested Revisions:

In the "Participants" subsection of the "Method" section, please add a few sentences to explicitly acknowledge that combining this sample was a pragmatic decision driven by statistical power considerations (i.e., small subgroup sample sizes), but that it is also a significant limitation.

In the "Discussion" and/or "Limitations" section, please provide a more in-depth discussion. Explicitly state that the model fit issues found in this "mixed-bag" sample can, in fact, be interpreted as further evidence of the profound cultural diversity within the Latinx community. This would adeptly turn a methodological weakness into an argument that supports the paper's central thesis.

2. Elaboration on the Limitations of Sample Representativeness

Observed Issue: The manuscript mentions in the "Limitations" section that the entire sample was drawn from Texas, but the potential impact of this is insufficiently discussed.

Suggested Revision: Please add 2-3 sentences here to elaborate on this limitation more specifically. For example, you could mention that "Texas's unique border culture and socio-political history may have shaped the cultural identity and value expression of local Latinx university students in a distinct way, which may differ from that of Latinx youth in other regions such as California, Florida, or the Northeast." This will give readers a clearer understanding of the generalizability boundaries of the study's findings.

3. Further Reflection on the Low Reliability of "Mainstream Values" Subscales

Observed Issue: The manuscript accurately reports the low reliability of some "Mainstream Values" subscales (e.g., "Independence and Self-Reliance") and compares it with the original scale development study.

Suggested Revision: To make the discussion more thought-provoking, I suggest adding a speculative explanation in the "Discussion" section. For example: "This low internal consistency might reflect that contemporary college students hold a more complex and ambivalent attitude toward mainstream values like 'independence' or 'competition.' They may endorse these values in some contexts but not in others, leading to inconsistent response patterns on related items." This would encourage deeper reflection from the reader.

4. Minor but Important Details

Impact of Language: Please add a sentence in the "Limitations" section acknowledging that "measurements were administered only in English," which could be a potential limitation, as the meaning of certain cultural constructs may have subtle differences for bilingual participants across languages.

Readability of Tables: Please double-check all tables (Tables 1-5). Ensure that all abbreviations (e.g., CFI, TLI, RMSEA, SRMR) are defined in the notes below each table to facilitate understanding for readers from outside the immediate field.

IV. Concluding Remarks

In summary, this is a well-conceived, rigorously executed, and important manuscript. The revision suggestions above are intended to help the authors further refine their arguments, enabling the paper to have an even greater scholarly impact upon publication. I am confident in this study's prospects for publication and look forward to reading the revised version.

Recommendation: Accept with Minor Revisions
