# Supplementary material for: Mexican American Cultural Values Scale: Considerations for college students
Source: PLoS One. 2026 Sep 1;21(9):e0354954. doi: 10.1371/journal.pone.0354954 (PMC13533370; doi:10.1371/journal.pone.0354954)
Supplement: Supplementary file 2 [file pone.0354954.s004.docx]

**Responses to Review Feedback**

**Response to Editor**

Thank you for providing us with the opportunity to prepare a revision of our manuscript entitled "Mexican American Cultural Values Scale: Considerations for College Students." (PONE-D-25-50366). We greatly appreciate the reviewer’s comments, which have helped us to improve the manuscript. We have addressed the reviewer’s suggestions below (each comment is pasted below in bold with our responses following), and hope that our efforts made the paper acceptable for publication

**Reviewer #1 Reviews and Responses**

**The similarity index is high; please revise and resubmit. I didn't feel comfortable reading the whole thing considering the similarity. Also, its abstract part is confusing with a high similarity index.**

We thank Reviewer #1 for their transparency regarding the manuscript’s similarity index. While the dataset and accompanying methodology have been used and reported previously (see citations), the manuscript presents an analysis of psychometric properties within a subsample of this larger dataset. In this regard, the results presented have not been published previously and are unique to PLOS One. We have also included a statement within the Methods section (Participants and Procedures) that provides the listed citations where the methodology has been presented recently, and included the following citations for transparency:

Citations:

Bautista, A., & Venta, A. (2024). Attachment security and somatization: The mediating role of emotion dysregulation in Latinx college students. *Journal of Affective Disorders*, 351, 165-171.

Walker, J. H., Cuervo, M., & Venta, A. (2022). Familismo neutralizes the effects of discrimination on suicide-related thoughts and behaviors in Latinx young adults*. Journal of Affective Disorders*, 315, 57-63. https://doi.org/10.1016/j.jad.2022.07.058

Venta, A., Long, T., Mercado, A., Garcini, L., & Cadenas, G. (2022). When the United States says you do not belong: Suicide-related thoughts and behaviors among immigrant young adults varying in immigration legal status. *Suicide & Life-Threatening Behavior,* 1-11, https://doi.org/10.1111/sltb.12871.

**Reviewer #2**

***Review 2.1 -* Thank you for the opportunity to review the manuscript, "Mexican American Cultural Values Scale: Considerations for College Students." This is a rigorous, timely, and highly important study that addresses a critical gap in the psychological assessment literature. The investigation into the psychometric properties of the widely used Mexican American Cultural Values Scale (MACVS) within a college student population is not only necessary but long overdue. The manuscript is well-written, logically structured, and presents its findings with clarity and conviction. Overall, this is an excellent piece of scholarship that I strongly recommend for publication, following minor revisions**

*Response 2.1 -* We thank Reviewer #2 for their positive remarks regarding the presented manuscript and for the recommended revisions. We ensured that all recommended revisions were integrated into the manuscript, and we believe they have greatly enhanced its integrity, rigor, and readability.

***Review - 2.2.* The findings themselves are poised to have a substantial impact. The evidence demonstrating the psychometric shortcomings of the MACVS in this population serves as a crucial "course correction" for the field. It will compel researchers to be more critical in their instrument selection and encourage the development of more culturally and generationally appropriate measures. Furthermore, the discussion section is a standout, skillfully weaving the technical findings into broader theoretical conversations about Latinx heterogeneity, the complexities of acculturation, and the role of critical consciousness in shaping cultural values. This elevates the paper from a purely technical report to a thought-provoking theoretical contribution.**

*Response 2.2 -* We thank Reviewer #2 for these positive remarks regarding our manuscript’s theoretical contributions.

***Review 2.3. -* Methodologically, the study is exemplary. The use of Confirmatory Factor Analysis (CFA) and multi-group measurement invariance testing is appropriate and expertly executed. I commend the authors for their transparent and honest reporting of methodological challenges, such as poor model fit and negative error variances. This level of rigor and transparency reflects a strong command of psychometric principles and is a model for the field.**

*Response 2.3 -* We again thank Reviewer #2 for their positive feedback on our methodological challenges, particularly our transparency in reporting poor model fit and negative error variances.

***Review 2.4* - The findings themselves are poised to have a substantial impact. The evidence demonstrating the psychometric shortcomings of the MACVS in this population serves as a crucial "course correction" for the field. It will compel researchers to be more critical in their instrument selection and encourage the development of more culturally and generationally appropriate measures. Furthermore, the discussion section is a standout, skillfully weaving the technical findings into broader theoretical conversations about Latinx heterogeneity, the complexities of acculturation, and the role of critical consciousness in shaping cultural values. This elevates the paper from a purely technical report to a thought-provoking theoretical contribution.**

*Response 2.4 -* We thank this reviewer for acknowledging our efforts to extend the manuscript's psychometric contributions to broader theoretical implications. We agree with the reviewer and hope that this research will encourage other scholars to think critically about instrument selection, laying the groundwork for future work examining the complexity underlying Latinx heterogeneity, acculturation, and critical consciousness.

***Review 2.5 -* My recommendations are intended to further strengthen what is already a very strong manuscript. These are minor points that can be addressed without altering the core of the study.**

*Response 2.5 -* We thank Reviewer #2 for outlining the following recommendations and are grateful for the time and effort devoted to providing thoughtful feedback to enhance the manuscript. Although characterized as “minor,” we believe that integrating these recommendations has contributed significantly to the manuscript’s flow, consistency, and overall rigor.

***Review 2.6*. - Regarding the "Non-Mexican" Sample: A key concern is the methodological tension created by combining participants from diverse national origins (Salvadoran, Puerto Rican, etc.) into a single "non-Mexican" group. This practice runs counter to the manuscript's central, and correct, assertion that the Latinx community is not a monolith. This high level of intra-group heterogeneity is a confounding factor that likely contributed to the poor model fit and failure of invariance tests. Recommendation: I suggest explicitly addressing this in two places. First, in the "Method" section, please add a brief note acknowledging that this consolidation was a pragmatic choice due to statistical power constraints but is a notable limitation. Second, in the "Discussion" and/or "Limitations" section, please expand on this point by arguing that these very findings of misfit within a "mixed" group can be interpreted as further empirical support for the heterogeneity of the Latinx population. This reframing turns a methodological limitation into a point that reinforces your central thesis.**

*Response 2.6. -* We thank this reviewer for the recommendation and wholeheartedly agree. We provide clarification regarding our decision to combine non-Mexican college students into a single group. Specifically, we have included a note in the Methods section and a new section in the Limitations to clarify that this decision was made for statistical power considerations, not to undermine our central argument – each country possesses rich intergenerational traditions and values that may diverge significantly. In the Limitation section, we also highlight the need to test the psychometric properties of the MACVS and other cultural values scales within each group.

***Review 2.7 -* Elaboration on Sample Representativeness: The manuscript correctly identifies the Texas-based sample as a limitation. Recommendation: To enhance this point, please briefly elaborate on why this matters. Adding a sentence or two explaining that Texas's unique socio-political and border culture may shape Latinx identity in ways distinct from other regions (e.g., California, Florida, the Northeast) would provide valuable context and clarify the boundaries of generalizability.**

*Response 2.7 -* We again thank the reviewer for this insight and recommendation, and agree with the need to contextualize our findings with respect to social, political, and geographical variables unique to young adult college students in Texas. We have elaborated on this section of our Limitations (p.30) to address the unique socio-political environment of Texas and the geographical proximity of border regions to Mexico, which can greatly shape how cultural values are passed down and diversified across Texas. Specifically, we call attention to 1) the prevalence of Mexican American descendants across various cities and 2) the close geographic proximity of Texas residents to Mexico, compared with other non-Mexican American groups, such as Colombia, Venezuela, and Chile, which are geographically distant. Additionally, we acknowledge that immigration and identity in the United States, especially among undocumented individuals, greatly influence perceived safety and retention of cultural values out of survival and resilience, further complicating the navigation of identity shifts as bicultural young adults.

***Response 2.8 -* Deeper Reflection on Low Reliability: The paper accurately reports the low reliability of several "Mainstream Values" subscales. Recommendation: To add another layer of depth to your discussion, please consider adding a speculative sentence about why this might be the case. For instance, you could suggest that contemporary college students may hold more ambivalent or context-dependent views on concepts like "independence" and "competition," leading to less consistent response patterns on these items.**

*Response 2.8 -* We agree with this reviewer and thank them for their recommendation. We elaborated on our Discussion section, “Comparisons with Previous Psychometric Evaluations of the MACVS,” which compares internal reliabilities with previous psychometric evaluations and highlights that reliabilities in the Mexican American college sample are higher than those in the Mainstream Values. We added two sentences to this section to illustrate that, while internal reliabilities are overall low, this prompts both a re-evaluation of the MACVS with respect to the Mainstream Value operationalization and an acknowledgment of the college-attendance context.

***Response 2.9 -* Language of Administration: Please add a brief mention in the "Limitations" section that the measures were administered only in English, acknowledging this as a potential limitation for bilingual participants.**

*Response 2.9 -* We agree with Reviewer #2 (and Reviewer #3) concerning the utilization of the MACVS for non-native or bilingual Spanish and English speakers. We have included a paragraph in the Limitations section (p.26) noting that the MACVS was administered in English, which might have been difficult for non-native English speakers and bilingual Spanish-English speakers to comprehend as deeply as native English speakers. While this is a significant and valid concern, slightly over half of our sample reported their native language as English (57.2%) across the total sample, and across the Mexican American (58.1%) and non-Mexican (50.3%) subsamples. Approximately 41.6% of Mexican Americans and 49.7% of non-Mexican subsamples reported English as their second language. We also now call for continued recognition of language fluency in the MACVS administration for English- and Spanish-speaking individuals.

***Review 2.10* - Table Clarity: Please conduct a final check of all tables to ensure that all statistical abbreviations (CFI, TLI, RMSEA, SRMR, etc.) are clearly defined in the table notes. This is a small detail that greatly improves readability for a broader audience.**

*Review 2.10 -* We have updated all tables and included statistical abbreviations as needed.

***Review 2.11 -* Conclusion: In conclusion, this is an excellent manuscript that makes a significant and valuable contribution to the field. The proposed revisions are minor and intended to further refine and strengthen its arguments. I am confident in this paper's suitability for publication and look forward to seeing the revised version in print.**

*Response 2.11 -* We sincerely thank the reviewer for the time and effort devoted to evaluating this manuscript. We believe that the reviewer’s recommendations have substantially improved the manuscript’s clarity and rigor. We hope that the revisions made have adequately addressed the concerns and suggestions raised.

**Reviewer #3 Review and Responses.**

**Review 3.1 - The present study investigates the psychometric functioning of the Mexican American Cultural Values Scale (MACVS) in Mexican American and non-Mexican Latinx college students and addresses a highly relevant question, given the widespread use of the instrument beyond its original developmental context. The manuscript demonstrates substantial analytic effort and covers many core psychometric steps. However, several additional analyses and clarifications would substantially strengthen the contribution and improve the interpretability of the findings.**

*Response 3.1 -* We thank Reviewer #3 for their time and effort in providing clear and constructive recommendations for improving the manuscript, particularly with regard to the reporting of MACVS’s psychometric properties and the clarification of the decision-making steps, analytic approach, and underlying logic that preceded our exploratory measurement invariance analyses between Mexican and non-Mexican Latinx students. We believe that the manuscript has improved substantially as a result of these recommendations.

***Review 3.2 -* The abstract would benefit from being made more explicitly psychometric in nature. At present, it describes the study aims and general conclusions but omits key quantitative information that is central to evaluating a measurement-focused manuscript. Including representative model fit indices (e.g., CFI, RMSEA, SRMR), reliability estimates, and a clear statement regarding the level at which measurement invariance failed would allow readers to immediately understand the magnitude and implications of the psychometric findings. Expanding the results portion of the abstract to reflect these statistical outcomes would better align it with the study’s methodological focus.**

*Response 3.2 -* We agree with Reviewer #3 regarding the need for more detailed psychometric information in the manuscript’s abstract, including the reporting of model fit indices and the extent of measurement failure. Accordingly, we revised the abstract to more clearly emphasize the manuscript's psychometric focus while adhering to the journal’s word limit. The revised abstract now includes additional detail on the model fit of the higher-order CFA model recommended by Knight et al. (2010), specifically reporting CFI, TLI, RMSEA, and SRMR indices, to better align with the study’s central hypothesis testing the higher-order correlated model among college-attending Mexican students.

***Review 3.3* - At present, the manuscript would benefit from a clearer and more explicit articulation of its primary objectives. It is not always evident whether the central goal is (a) to replicate the original MACVS factor structure in college students, (b) to evaluate the psychometric adequacy of the higher-order model in this population, or (c) to assess cross-group comparability between Mexican American and non-Mexican Latinx students. Explicitly distinguishing these aims (both in the Introduction and when motivating each analytic step) would help readers better understand the logic of the analyses and the implications of the findings.**

*Response 3.3 -* We have reframed the manuscript to clarify the decision process, while ensuring that the central hypothesis – CFA testing of the MACVS for college-attending Mexican American students is reiterated throughout. We also clarified that tests, including the MACVS for non-Mexican Latinx college students and measurement invariance, are now grouped under an “Exploratory Analysis section” due to the broad use of the MACVS beyond Mexican Americans in recent literature.

***Review 3.4 -* Given the consistent lack of acceptable fit across confirmatory models and the recurrent issues with non-positive definite matrices, the manuscript would benefit from incorporating an explicit exploratory phase. Once the originally hypothesized factor structure fails to replicate, continuing exclusively with confirmatory models may obscure rather than clarify the nature of the measurement problem. Conducting an exploratory factor analysis or an exploratory structural equation modeling (ESEM) approach within the college student sample would help determine whether an alternative latent structure better represents cultural values in this population, or whether misfit reflects item-level weaknesses rather than structural failure.**

*Response 3.4 -* We thank this reviewer for their recommendation to test and have added the results of the exploratory factor analyses across the entire sample, including Mexican American and non-Mexican college students, to examine whether an alternative latent structure better represents the data and to evaluate item-level weaknesses. Results of these analyses are now summarized in the “Exploratory Analysis” section of the manuscript (p. 20), and detail the retention of six factors with an absolute good fit RMSEA = .048 (90% CI [.045, .051]), RMSR = .03, TLI = .896, and accounted for 50% of total item variance. However, closer inspection of the pattern matrix revealed both structural preservation and reorganization relative to the original model, particularly with regard to *Respect* items loading jointly with *Familism-Referent* indicators, as well as the emergence of a sixth factor characterized by bipolar structure with indicators from *Traditional Gender Role* endorsement and *Independence/Self-Reliance*. The implications of this EFA are also included within the Discussion section.

***Review 3.5 -* Reliability assessment could be strengthened by moving beyond Cronbach’s alpha alone. Given the latent-variable framework adopted throughout the manuscript and the presence of relatively short subscales, the inclusion of McDonald’s omega or composite reliability estimates at the factor level would provide a more appropriate and informative evaluation of internal consistency. This would also align the reliability evidence more closely with the CFA results and help clarify whether psychometric problems stem from measurement precision or from model misspecification (expecially for subscales compsed by 4-5 items).**

*Response 3.5 -* We thank the reviewer for this thoughtful suggestion. We agree that omega and composite reliability would provide useful information; however, we reported Cronbach’s alpha to maintain consistency with prior research using this measure. Additionally, we aimed to avoid introducing analyses that would expand the scope of the manuscript beyond its original design, as the paper was invited for revision rather than major changes. To address this concern, we have now included composite reliability estimates in the revised Table 2 and on page 15. These estimates indicate that reliability was generally consistent and acceptable across the Mexican American, non-Mexican, and total samples. Reliability coefficients ranged from low reliability for the *Independence/Self-Reliance* subscale to strong reliability for the *Religiosity* subscale.

***Review 3.6 -* With respect to measurement invariance, the analysis currently stops at configural non-invariance. While this is methodologically defensible, additional steps could yield important diagnostic information. Testing partial invariance or identifying specific items contributing to non-invariance would help clarify whether the MACVS fails globally across groups or whether non-equivalence is localized to specific dimensions (e.g., Mainstream Values). This information would be especially valuable for researchers who continue to use selected MACVS subscales rather than the full higher-order structure.**

*Response 3.6 -* Thank you for this suggestion. We agree that including information on sources of noninvariance can provide important diagnostic information. In response, we conducted follow-up analyses using the converged configural model to examine cross-group differences in standardized factor loadings. In particular, we compared item-level loadings across groups to determine whether discrepancies were global or localized. Results, now included on pp. 20-21, indicated that loading differences were mainly in the subscales of Independence & Self-Reliance and Traditional Gender Roles subscales. The majority of other items demonstrated small cross-group differences.

***Review 3.7* - The interpretation of the invariance results would benefit from more explicit consideration of group-size imbalance. The Mexican American and non-Mexican Latinx samples differ substantially in size, a condition known to affect multigroup CFA stability, convergence, and fit statistics, particularly in higher-order models. Addressing this issue analytically or through sensitivity analyses would strengthen confidence in the conclusion that invariance failure reflects substantive group differences rather than methodological artifacts.**

*Response 3.7 -* We agree with Reviewer #3's concerns regarding group sizes and acknowledge that group-size imbalance can affect the fit of multigroup CFA models, particularly higher-order models. To evaluate whether unequal group sizes influenced the multigroup CFA results, we conducted a sensitivity analysis using a balanced subsample (*n* = 154). The configural multi-group model estimated in the balanced sample continued to demonstrate inadequate fit (CFI = .803, TLI = .788, RMSEA = .072, SRMR = .088), consistent with the pattern observed in the full sample. Thus, the lack of configural invariance does not appear to be solely due to group-size imbalance. We include these findings on p.19 of the manuscript.

***Review 3.8 -* The exclusive administration of the MACVS in English warrants more direct consideration within the psychometric argument. Because the scale assesses culturally embedded values, language of administration may influence item interpretation, especially among participants who are non–U.S. citizens or who may primarily use Spanish in family contexts. Explicitly examining or discussing the role of language in shaping factor structure and item functioning would enhance the construct validity evaluation.**

*Response 3.8 -* Please see *Response 2.9.* We have also included a proportion of Mexican and non-Mexican college students who speak Spanish fluently or are native Spanish speakers.

***Review 3.9 -* The results section would benefit from greater synthesis across the numerous models tested. Explicitly summarizing which components of the MACVS perform adequately and which do not, rather than presenting misfit model by model, would improve clarity and help readers focus on the substantive psychometric conclusions.del, would improve clarity and help readers focus on the substantive psychometric conclusions.**

*Response 3.9 -* We thank this reviewer for the recommendation and agree that the results section would benefit greatly from a summary of the tested models. A summary within the results section, at the end of the Primary Analyses and before the Exploratory Analyses sections, now provides an overview of all tested models and outlines similarities between the first-ordered models that failed to converge and the less-than-adequate model fit indices of the two higher-ordered models.

***Review 3.10 -* Finally, the Discussion would be strengthened by more clearly linking the observed psychometric difficulties to the sociocultural and developmental context of contemporary college students. Rather than framing the findings primarily as evidence of poor model fit, the manuscript could more explicitly explore whether generational change, increased acculturation, and shifting norms—particularly regarding gender roles and individualism—necessitate reconceptualization or updating of the MACVS for young adult populations. This reframing would allow the results to be interpreted not only as a measurement failure, but also as informative evidence about the evolving nature of cultural values.**

*Response 3.10 -* We agree with the recommendation from Reviewer #3. As we previously discussed in *Response 2.7*, we now further elaborate on the implications of model misfit in the Discussion section (pp. 20 - 21), elaborating on possible generational changes in cultural value transmission, identity shifts, and norm development that uniquely shape college-attending Latinx students' endorsement of Traditional Values. We also discuss the implications of model misfit with a gendered norms lens, specifically highlighting that college attendance may bring into sharper focus the financial and social costs associated with it, and that these costs may be greater for Latinas who experience role conflict between college-attendance, potential career selection, and maternal duties to care for husbands and children.
