# Supplementary material for: Mexican American Cultural Values Scale: Considerations for college students
Source: PLoS One. 2026 Sep 1;21(9):e0354954. doi: 10.1371/journal.pone.0354954 (PMC13533370; doi:10.1371/journal.pone.0354954)
Supplement: Peer review history

# Peer review: Decision Letter 0

**DOI:** 10.1371/journal.pone.0354954.r001

**Authors:** Claudia Noemi González Brambila

15 Jan 2026

Dear Dr. Vázquez,

Thank you for submitting your manuscript to PLOS ONE. After careful consideration, we feel that it has merit but does not fully meet PLOS ONE’s publication criteria as it currently stands. Therefore, we invite you to submit a revised version of the manuscript that addresses the points raised during the review process.

Please submit your revised manuscript by Mar 01 2026 11:59PM. If you will need more time than this to complete your revisions, please reply to this message or contact the journal office at plosone@plos.org. When you're ready to submit your revision, log on to https://www.editorialmanager.com/pone/ and select the 'Submissions Needing Revision' folder to locate your manuscript file.

- A letter that responds to each point raised by the academic editor and reviewer(s). You should upload this letter as a separate file labeled 'Response to Reviewers'.
- A marked-up copy of your manuscript that highlights changes made to the original version. You should upload this as a separate file labeled 'Revised Manuscript with Track Changes'.
- An unmarked version of your revised paper without tracked changes. You should upload this as a separate file labeled 'Manuscript'.

If you would like to make changes to your financial disclosure, please include your updated statement in your cover letter. Guidelines for resubmitting your figure files are available below the reviewer comments at the end of this letter.

If applicable, we recommend that you deposit your laboratory protocols in protocols.io to enhance the reproducibility of your results. Protocols.io assigns your protocol its own identifier (DOI) so that it can be cited independently in the future. For instructions see: https://journals.plos.org/plosone/s/submission-guidelines#loc-laboratory-protocols. Additionally, PLOS ONE offers an option for publishing peer-reviewed Lab Protocol articles, which describe protocols hosted on protocols.io. Read more information on sharing protocols at https://plos.org/protocols?utm_medium=editorial-email&utm_source=authorletters&utm_campaign=protocols.

We look forward to receiving your revised manuscript.

Kind regards,

Claudia Noemi González Brambila, Ph.D.

Academic Editor

PLOS One

Journal Requirements:

When submitting your revision, we need you to address these additional requirements.

1. Please ensure that your manuscript meets PLOS ONE's style requirements, including those for file naming. The PLOS ONE style templates can be found at

https://journals.plos.org/plosone/s/file?id=wjVg/PLOSOne_formatting_sample_main_body.pdf and

https://journals.plos.org/plosone/s/file?id=ba62/PLOSOne_formatting_sample_title_authors_affiliations.pdf.

2. Please provide additional details regarding participant consent. In the ethics statement in the Methods and online submission information, please ensure that you have specified (1) whether consent was informed and (2) what type you obtained (for instance, written or verbal, and if verbal, how it was documented and witnessed). If your study included minors, state whether you obtained consent from parents or guardians. If the need for consent was waived by the ethics committee, please include this information.

If you are reporting a retrospective study of medical records or archived samples, please ensure that you have discussed whether all data were fully anonymized before you accessed them and/or whether the IRB or ethics committee waived the requirement for informed consent. If patients provided informed written consent to have data from their medical records used in research, please include this information.

3. In the online submission form, you indicated that [This study includes members of a legally and socially vulnerable population who face ongoing risk of persecution and harm. De-identified data will be provided based on reasonable request to confirm study findings.].

All PLOS journals now require all data underlying the findings described in their manuscript to be freely available to other researchers, either 1. In a public repository, 2. Within the manuscript itself, or 3. Uploaded as supplementary information.

This policy applies to all data except where public deposition would breach compliance with the protocol approved by your research ethics board. If your data cannot be made publicly available for ethical or legal reasons (e.g., public availability would compromise patient privacy), please explain your reasons on resubmission and your exemption request will be escalated for approval.

4. If the reviewer comments include a recommendation to cite specific previously published works, please review and evaluate these publications to determine whether they are relevant and should be cited. There is no requirement to cite these works unless the editor has indicated otherwise.

[Note: HTML markup is below. Please do not edit.]

Reviewers' comments:

Reviewer's Responses to Questions

**Comments to the Author**

1. Is the manuscript technically sound, and do the data support the conclusions?

Reviewer #1: Partly

Reviewer #2: Partly

Reviewer #3: Partly

**********

2. Has the statistical analysis been performed appropriately and rigorously?

Reviewer #1: No

Reviewer #2: Yes

Reviewer #3: No

**********

3. Have the authors made all data underlying the findings in their manuscript fully available?

The PLOS Data policy

Reviewer #1: Yes

Reviewer #2: Yes

Reviewer #3: No

**********

4. Is the manuscript presented in an intelligible fashion and written in standard English?

Reviewer #1: Yes

Reviewer #2: Yes

Reviewer #3: Yes

**********

Reviewer #1: The similarity index is high; please revise and resubmit. I didn't feel comfortable reading the whole thing considering the similarity. Also, its abstract part is confusing with a high similarity index.

Reviewer #2: Reviewer Comments to the Author

Thank you for the opportunity to review the manuscript, "Mexican American Cultural Values Scale: Considerations for College Students." This is a rigorous, timely, and highly important study that addresses a critical gap in the psychological assessment literature. The investigation into the psychometric properties of the widely used Mexican American Cultural Values Scale (MACVS) within a college student population is not only necessary but long overdue. The manuscript is well-written, logically structured, and presents its findings with clarity and conviction. Overall, this is an excellent piece of scholarship that I strongly recommend for publication, following minor revisions.

Major Strengths:

The study's primary strength lies in its significant originality and practical importance. It tackles the often-overlooked issue of using established psychological measures in new, untested populations—a practice that can lead to flawed research conclusions. By focusing on college students, the authors address a demographic of immense interest to researchers across various disciplines.

Methodologically, the study is exemplary. The use of Confirmatory Factor Analysis (CFA) and multi-group measurement invariance testing is appropriate and expertly executed. I commend the authors for their transparent and honest reporting of methodological challenges, such as poor model fit and negative error variances. This level of rigor and transparency reflects a strong command of psychometric principles and is a model for the field.

The findings themselves are poised to have a substantial impact. The evidence demonstrating the psychometric shortcomings of the MACVS in this population serves as a crucial "course correction" for the field. It will compel researchers to be more critical in their instrument selection and encourage the development of more culturally and generationally appropriate measures. Furthermore, the discussion section is a standout, skillfully weaving the technical findings into broader theoretical conversations about Latinx heterogeneity, the complexities of acculturation, and the role of critical consciousness in shaping cultural values. This elevates the paper from a purely technical report to a thought-provoking theoretical contribution.

Suggestions for Revision:

My recommendations are intended to further strengthen what is already a very strong manuscript. These are minor points that can be addressed without altering the core of the study.

Regarding the "Non-Mexican" Sample: A key concern is the methodological tension created by combining participants from diverse national origins (Salvadoran, Puerto Rican, etc.) into a single "non-Mexican" group. This practice runs counter to the manuscript's central, and correct, assertion that the Latinx community is not a monolith. This high level of intra-group heterogeneity is a confounding factor that likely contributed to the poor model fit and failure of invariance tests.

Recommendation: I suggest explicitly addressing this in two places. First, in the "Method" section, please add a brief note acknowledging that this consolidation was a pragmatic choice due to statistical power constraints but is a notable limitation. Second, in the "Discussion" and/or "Limitations" section, please expand on this point by arguing that these very findings of misfit within a "mixed" group can be interpreted as further empirical support for the heterogeneity of the Latinx population. This reframing turns a methodological limitation into a point that reinforces your central thesis.

Elaboration on Sample Representativeness: The manuscript correctly identifies the Texas-based sample as a limitation.

Recommendation: To enhance this point, please briefly elaborate on why this matters. Adding a sentence or two explaining that Texas's unique socio-political and border culture may shape Latinx identity in ways distinct from other regions (e.g., California, Florida, the Northeast) would provide valuable context and clarify the boundaries of generalizability.

Deeper Reflection on Low Reliability: The paper accurately reports the low reliability of several "Mainstream Values" subscales.

Recommendation: To add another layer of depth to your discussion, please consider adding a speculative sentence about why this might be the case. For instance, you could suggest that contemporary college students may hold more ambivalent or context-dependent views on concepts like "independence" and "competition," leading to less consistent response patterns on these items.

Minor Details for Enhanced Rigor:

Language of Administration: Please add a brief mention in the "Limitations" section that the measures were administered only in English, acknowledging this as a potential limitation for bilingual participants.

Table Clarity: Please conduct a final check of all tables to ensure that all statistical abbreviations (CFI, TLI, RMSEA, SRMR, etc.) are clearly defined in the table notes. This is a small detail that greatly improves readability for a broader audience.

Conclusion:

In conclusion, this is an excellent manuscript that makes a significant and valuable contribution to the field. The proposed revisions are minor and intended to further refine and strengthen its arguments. I am confident in this paper's suitability for publication and look forward to seeing the revised version in print.

No concerns regarding dual publication, research ethics, or publication ethics were noted.

Reviewer #3: The present study investigates the psychometric functioning of the Mexican American Cultural Values Scale (MACVS) in Mexican American and non-Mexican Latinx college students and addresses a highly relevant question, given the widespread use of the instrument beyond its original developmental context. The manuscript demonstrates substantial analytic effort and covers many core psychometric steps. However, several additional analyses and clarifications would substantially strengthen the contribution and improve the interpretability of the findings.

1) The abstract would benefit from being made more explicitly psychometric in nature. At present, it describes the study aims and general conclusions but omits key quantitative information that is central to evaluating a measurement-focused manuscript. Including representative model fit indices (e.g., CFI, RMSEA, SRMR), reliability estimates, and a clear statement regarding the level at which measurement invariance failed would allow readers to immediately understand the magnitude and implications of the psychometric findings. Expanding the results portion of the abstract to reflect these statistical outcomes would better align it with the study’s methodological focus.

2) At present, the manuscript would benefit from a clearer and more explicit articulation of its primary objectives. It is not always evident whether the central goal is (a) to replicate the original MACVS factor structure in college students, (b) to evaluate the psychometric adequacy of the higher-order model in this population, or (c) to assess cross-group comparability between Mexican American and non-Mexican Latinx students. Explicitly distinguishing these aims (both in the Introduction and when motivating each analytic step) would help readers better understand the logic of the analyses and the implications of the findings.

3) Given the consistent lack of acceptable fit across confirmatory models and the recurrent issues with non-positive definite matrices, the manuscript would benefit from incorporating an explicit exploratory phase. Once the originally hypothesized factor structure fails to replicate, continuing exclusively with confirmatory models may obscure rather than clarify the nature of the measurement problem. Conducting an exploratory factor analysis or an exploratory structural equation modeling (ESEM) approach within the college student sample would help determine whether an alternative latent structure better represents cultural values in this population, or whether misfit reflects item-level weaknesses rather than structural failure.

4) Reliability assessment could be strengthened by moving beyond Cronbach’s alpha alone. Given the latent-variable framework adopted throughout the manuscript and the presence of relatively short subscales, the inclusion of McDonald’s omega or composite reliability estimates at the factor level would provide a more appropriate and informative evaluation of internal consistency. This would also align the reliability evidence more closely with the CFA results and help clarify whether psychometric problems stem from measurement precision or from model misspecification (expecially for subscales compsed by 4-5 items).

5) With respect to measurement invariance, the analysis currently stops at configural non-invariance. While this is methodologically defensible, additional steps could yield important diagnostic information. Testing partial invariance or identifying specific items contributing to non-invariance would help clarify whether the MACVS fails globally across groups or whether non-equivalence is localized to specific dimensions (e.g., Mainstream Values). This information would be especially valuable for researchers who continue to use selected MACVS subscales rather than the full higher-order structure.

6) The interpretation of the invariance results would benefit from more explicit consideration of group-size imbalance. The Mexican American and non-Mexican Latinx samples differ substantially in size, a condition known to affect multigroup CFA stability, convergence, and fit statistics, particularly in higher-order models. Addressing this issue analytically or through sensitivity analyses would strengthen confidence in the conclusion that invariance failure reflects substantive group differences rather than methodological artifacts.

7) The exclusive administration of the MACVS in English warrants more direct consideration within the psychometric argument. Because the scale assesses culturally embedded values, language of administration may influence item interpretation, especially among participants who are non–U.S. citizens or who may primarily use Spanish in family contexts. Explicitly examining or discussing the role of language in shaping factor structure and item functioning would enhance the construct validity evaluation.

8) The results section would benefit from greater synthesis across the numerous models tested. Explicitly summarizing which components of the MACVS perform adequately and which do not, rather than presenting misfit model by model, would improve clarity and help readers focus on the substantive psychometric conclusions.

9) Finally, the Discussion would be strengthened by more clearly linking the observed psychometric difficulties to the sociocultural and developmental context of contemporary college students. Rather than framing the findings primarily as evidence of poor model fit, the manuscript could more explicitly explore whether generational change, increased acculturation, and shifting norms—particularly regarding gender roles and individualism—necessitate reconceptualization or updating of the MACVS for young adult populations. This reframing would allow the results to be interpreted not only as a measurement failure, but also as informative evidence about the evolving nature of cultural values.

Taken together, these additions would significantly enhance the manuscript’s psychometric rigor and clarify its implications for both researchers and clinicians who rely on the MACVS. Addressing these points would strengthen the study’s contribution as a methodological reference for cultural values assessment in Latinx college populations.

**********

what does this mean?). If published, this will include your full peer review and any attached files.

If you choose “no”, your identity will remain anonymous but your review may still be made public.

**Do you want your identity to be public for this peer review?**  For information about this choice, including consent withdrawal, please see our Privacy Policy

Reviewer #1: No

Reviewer #2: No

Reviewer #3: No

**********

[NOTE: If reviewer comments were submitted as an attachment file, they will be attached to this email and accessible via the submission site. Please log into your account, locate the manuscript record, and check for the action link "View Attachments". If this link does not appear, there are no attachment files.]

To ensure your figures meet our technical requirements, please review our figure guidelines: https://journals.plos.org/plosone/s/figures

You may also use PLOS’s free figure tool, NAAS, to help you prepare publication quality figures: https://journals.plos.org/plosone/s/figures#loc-tools-for-figure-preparation.

NAAS will assess whether your figures meet our technical requirements by comparing each figure against our figure specifications.

Attachment Submitted filename: Accept with Minor Revisions.docx

# Author comment: Author response to Decision Letter 1

**DOI:** 10.1371/journal.pone.0354954.r002

5 Mar 2026

Responses to Review Feedback

Response to Editor

Thank you for providing us with the opportunity to prepare a revision of our manuscript entitled "Mexican American Cultural Values Scale: Considerations for College Students." (PONE-D-25-50366). We greatly appreciate the reviewer’s comments, which have helped us to improve the manuscript. We have addressed the reviewer’s suggestions below (each comment is pasted below in bold with our responses following), and hope that our efforts made the paper acceptable for publication

Reviewer #1 Reviews and Responses

The similarity index is high; please revise and resubmit. I didn't feel comfortable reading the whole thing considering the similarity. Also, its abstract part is confusing with a high similarity index.

We thank Reviewer #1 for their transparency regarding the manuscript’s similarity index. While the dataset and accompanying methodology have been used and reported previously (see citations), the manuscript presents an analysis of psychometric properties within a subsample of this larger dataset. In this regard, the results presented have not been published previously and are unique to PLOS One. We have also included a statement within the Methods section (Participants and Procedures) that provides the listed citations where the methodology has been presented recently, and included the following citations for transparency:

Citations:

Bautista, A., & Venta, A. (2024). Attachment security and somatization: The mediating role of emotion dysregulation in Latinx college students. Journal of Affective Disorders, 351, 165-171.

Walker, J. H., Cuervo, M., & Venta, A. (2022). Familismo neutralizes the effects of discrimination on suicide-related thoughts and behaviors in Latinx young adults. Journal of Affective Disorders, 315, 57-63. https://doi.org/10.1016/j.jad.2022.07.058

Venta, A., Long, T., Mercado, A., Garcini, L., & Cadenas, G. (2022). When the United States says you do not belong: Suicide-related thoughts and behaviors among immigrant young adults varying in immigration legal status. Suicide & Life-Threatening Behavior, 1-11, https://doi.org/10.1111/sltb.12871.

Reviewer #2

Review 2.1 - Thank you for the opportunity to review the manuscript, "Mexican American Cultural Values Scale: Considerations for College Students." This is a rigorous, timely, and highly important study that addresses a critical gap in the psychological assessment literature. The investigation into the psychometric properties of the widely used Mexican American Cultural Values Scale (MACVS) within a college student population is not only necessary but long overdue. The manuscript is well-written, logically structured, and presents its findings with clarity and conviction. Overall, this is an excellent piece of scholarship that I strongly recommend for publication, following minor revisions

Response 2.1 - We thank Reviewer #2 for their positive remarks regarding the presented manuscript and for the recommended revisions. We ensured that all recommended revisions were integrated into the manuscript, and we believe they have greatly enhanced its integrity, rigor, and readability.

Review - 2.2. The findings themselves are poised to have a substantial impact. The evidence demonstrating the psychometric shortcomings of the MACVS in this population serves as a crucial "course correction" for the field. It will compel researchers to be more critical in their instrument selection and encourage the development of more culturally and generationally appropriate measures. Furthermore, the discussion section is a standout, skillfully weaving the technical findings into broader theoretical conversations about Latinx heterogeneity, the complexities of acculturation, and the role of critical consciousness in shaping cultural values. This elevates the paper from a purely technical report to a thought-provoking theoretical contribution.

Response 2.2 - We thank Reviewer #2 for these positive remarks regarding our manuscript’s theoretical contributions.

Review 2.3. - Methodologically, the study is exemplary. The use of Confirmatory Factor Analysis (CFA) and multi-group measurement invariance testing is appropriate and expertly executed. I commend the authors for their transparent and honest reporting of methodological challenges, such as poor model fit and negative error variances. This level of rigor and transparency reflects a strong command of psychometric principles and is a model for the field.

Response 2.3 - We again thank Reviewer #2 for their positive feedback on our methodological challenges, particularly our transparency in reporting poor model fit and negative error variances.

Review 2.4 - The findings themselves are poised to have a substantial impact. The evidence demonstrating the psychometric shortcomings of the MACVS in this population serves as a crucial "course correction" for the field. It will compel researchers to be more critical in their instrument selection and encourage the development of more culturally and generationally appropriate measures. Furthermore, the discussion section is a standout, skillfully weaving the technical findings into broader theoretical conversations about Latinx heterogeneity, the complexities of acculturation, and the role of critical consciousness in shaping cultural values. This elevates the paper from a purely technical report to a thought-provoking theoretical contribution.

Response 2.4 - We thank this reviewer for acknowledging our efforts to extend the manuscript's psychometric contributions to broader theoretical implications. We agree with the reviewer and hope that this research will encourage other scholars to think critically about instrument selection, laying the groundwork for future work examining the complexity underlying Latinx heterogeneity, acculturation, and critical consciousness.

Review 2.5 - My recommendations are intended to further strengthen what is already a very strong manuscript. These are minor points that can be addressed without altering the core of the study.

Response 2.5 - We thank Reviewer #2 for outlining the following recommendations and are grateful for the time and effort devoted to providing thoughtful feedback to enhance the manuscript. Although characterized as “minor,” we believe that integrating these recommendations has contributed significantly to the manuscript’s flow, consistency, and overall rigor.

Review 2.6. - Regarding the "Non-Mexican" Sample: A key concern is the methodological tension created by combining participants from diverse national origins (Salvadoran, Puerto Rican, etc.) into a single "non-Mexican" group. This practice runs counter to the manuscript's central, and correct, assertion that the Latinx community is not a monolith. This high level of intra-group heterogeneity is a confounding factor that likely contributed to the poor model fit and failure of invariance tests. Recommendation: I suggest explicitly addressing this in two places. First, in the "Method" section, please add a brief note acknowledging that this consolidation was a pragmatic choice due to statistical power constraints but is a notable limitation. Second, in the "Discussion" and/or "Limitations" section, please expand on this point by arguing that these very findings of misfit within a "mixed" group can be interpreted as further empirical support for the heterogeneity of the Latinx population. This reframing turns a methodological limitation into a point that reinforces your central thesis.

Response 2.6. - We thank this reviewer for the recommendation and wholeheartedly agree. We provide clarification regarding our decision to combine non-Mexican college students into a single group. Specifically, we have included a note in the Methods section and a new section in the Limitations to clarify that this decision was made for statistical power considerations, not to undermine our central argument – each country possesses rich intergenerational traditions and values that may diverge significantly. In the Limitation section, we also highlight the need to test the psychometric properties of the MACVS and other cultural values scales within each group.

Review 2.7 - Elaboration on Sample Representativeness: The manuscript correctly identifies the Texas-based sample as a limitation. Recommendation: To enhance this point, please briefly elaborate on why this matters. Adding a sentence or two explaining that Texas's unique socio-political and border culture may shape Latinx identity in ways distinct from other regions (e.g., California, Florida, the Northeast) would provide valuable context and clarify the boundaries of generalizability.

Response 2.7 - We again thank the reviewer for this insight and recommendation, and agree with the need to contextualize our findings with respect to social, political, and geographical variables unique to young adult college students in Texas. We have elaborated on this section of our Limitations (p.30) to address the unique socio-political environment of Texas and the geographical proximity of border regions to Mexico, which can greatly shape how cultural values are passed down and diversified across Texas. Specifically, we call attention to 1) the prevalence of Mexican American descendants across various cities and 2) the close geographic proximity of Texas residents to Mexico, compared with other non-Mexican American groups, such as Colombia, Venezuela, and Chile, which are geographically distant. Additionally, we acknowledge that immigration and identity in the United States, especially among undocumented individuals, greatly influence perceived safety and retention of cultural values out of survival and resilience, further complicating the navigation of identity shifts as bicultural young adults.

Response 2.8 - Deeper Reflection on Low Reliability: The paper accurately reports the low reliability of several "Mainstream Values" subscales. Recommendation: To add another layer of depth to your discussion, please consider adding a speculative sentence about why this might be the case. For instance, you could suggest that contemporary college students may hold more ambivalent or context-dependent views on concepts like "independence" and "competition," leading to less consistent response patterns on these items.

Response 2.8 - We agree with this reviewer and thank them for their recommendation. We elaborated on our Discussion section, “Comparisons with Previous Psychometric Evaluations of the MACVS,” which compares internal reliabilities with previous psychometric evaluations and highlights that reliabilities in the Mexican American college sample are higher than those in the Mainstream Values. We added two sentences to this section to illustrate that, while internal reliabilities are overall low, this prompts both a re-evaluation of the MACVS with respect to the Mainstream Value operationalization and an acknowledgment of the college-attendance context.

Response 2.9 - Language of Administration: Please add a brief mention in the "Limitations" section that the measures were administered only in English, acknowledging this as a potential limitation for bilingual participants.

Response 2.9 - We agree with Reviewer #2 (and Reviewer #3) concerning the utilization of the MACVS for non-native or bilingual Spanish and English speakers. We have included a paragraph in the Limitations section (p.26) noting that the MACVS was administered in English, which might have been difficult for non-native English speakers and bilingual Spanish-English speakers to comprehend as deeply as native English speakers. While this is a significant and valid concern, slightly over half of our sample reported their native language as English (57.2%) across the total sample, and across the Mexican American (58.1%) and non-Mexican (50.3%) subsamples. Approximately 41.6% of Mexican Americans and 49.7% of non-Mexican subsamples reported English as their second language. We also now call for continued recognition of language fluency in the MACVS administration for English- and Spanish-speaking individuals.

Review 2.10 - Table Clarity: Please conduct a final check of all tables to ensure that all statistical abbreviations (CFI, TLI, RMSEA, SRMR, etc.) are clearly defined in the table notes. This is a small detail that greatly improves readability for a broader audience.

Review 2.10 - We have updated all tables and included statistical abbreviations as needed.

Review 2.11 - Conclusion: In conclusion, this is an excellent manuscript that makes a significant and valuable contribution to the field. The proposed revisions are minor and intended to further refine and strengthen its arguments. I am confident in this paper's suitability for publication and look forward to seeing the revised version in print.

Response 2.11 - We sincerely thank the reviewer for the time and effort devoted to evaluating this manuscript. We believe that the reviewer’s recommendations have substantially improved the manuscript’s clarity and rigor. We hope that the revisions made have adequately addressed the concerns and suggestions raised.

Reviewer #3 Review and Responses.

Review 3.1 - The present study investigates the psychometric functioning of the Mexican American Cultural Values Scale (MACVS) in Mexican American and non-Mexican Latinx college students and addresses a highly relevant question, given the widespread use of the instrument beyond its original developmental context. The manuscript demonstrates substantial analytic effort and covers many core psychometric steps. However, several additional analyses and clarifications would substantially strengthen the contribution and improve the interpretability of the findings.

Response 3.1 - We thank Reviewer #3 for their time and effort in providing clear and constructive recommendations for improving the manuscript, particularly with regard to the reporting of MACVS’s psychometric properties and the clarification of the decision-making steps, analytic approach, and underlying logic that preceded our exploratory measurement invariance analyses between Mexican and non-Mexican Latinx students. We believe that the manuscript has improved substantially as a result of these recommendations.

Review 3.2 - The abstract would benefit from being made more explicitly psychometric in nature. At present, it describes the study aims and general conclusions but omits key quantitative information that is central to evaluating a measurement-focused manuscript. Including representative model fit indices (e.g., CFI, RMSEA, SRMR), reliability estimates, and a clear statement regarding the level at which measurement invariance failed would allow readers to immediately understand the magnitude and implications of the psychometric findings. Expanding the results portion of the abstract to reflect these statistical outcomes would better align it with the study’s methodological focus.

Response 3.2 - We agree with Reviewer #3 regarding the need for more detailed psychometric information in the manuscript’s abstract, including the reporting of model fit indices and the extent of measurement failure. Accordingly, we revised the abstract to more clearly emphasize the manuscript's psychometric focus while adhering to the journal’s word limit. The revised abstract now includes additional detail on the model fit of the higher-order CFA model recommended by Knight et al. (2010), specifically reporting CFI, TLI, RMSEA, and SRMR indices, to better align with the study’s central hypothesis testing the higher-order correlated model among college-attending Mexican students.

Review 3.3 - At present, the manuscript would benefit from a clearer and more explicit articulation of its primary objectives. It is not always evident whether the central goal is (a) to replicate the original MACVS factor structure in college students, (b) to evaluate the psychometric adequacy of the higher-order model in this population, or (c) to assess cross-group comparability between Mexican American and non-Mexican Latinx students. Explicitly distinguishing these aims (both in the Introduction and when motivating each analytic step) would help readers better understand the logic of the analyses and the implications

Attachment Submitted filename: Responses to Reviewers.docx

# Peer review: Decision Letter 1

**DOI:** 10.1371/journal.pone.0354954.r003

**Authors:** Claudia Noemi González Brambila

15 Jul 2026

Mexican American Cultural Values Scale: Considerations for College Students

PONE-D-25-50366R1

Dear Dr. Vázquez,

We’re pleased to inform you that your manuscript has been judged scientifically suitable for publication and will be formally accepted for publication once it meets all outstanding technical requirements.

Within one week, you’ll receive an e-mail detailing the required amendments. When these have been addressed, you’ll receive a formal acceptance letter and your manuscript will be scheduled for publication.

An invoice will be generated when your article is formally accepted. Please note, if your institution has a publishing partnership with PLOS and your article meets the relevant criteria, all or part of your publication costs will be covered. Please make sure your user information is up-to-date by logging into Editorial Manager at Editorial Manager® and clicking the ‘Update My Information' link at the top of the page. For questions related to billing, please contact billing support.

If your institution or institutions have a press office, please notify them about your upcoming paper to help maximize its impact. If they’ll be preparing press materials, please inform our press team as soon as possible -- no later than 48 hours after receiving the formal acceptance. Your manuscript will remain under strict press embargo until 2 pm Eastern Time on the date of publication. For more information, please contact onepress@plos.org.

Kind regards,

Claudia Noemi González Brambila, Ph.D.

Academic Editor

PLOS One

Additional Editor Comments (optional):

Reviewers' comments:

Reviewer's Responses to Questions

**Comments to the Author**

Reviewer #1: All comments have been addressed

Reviewer #3: All comments have been addressed

**********

2. Is the manuscript technically sound, and do the data support the conclusions?

Reviewer #1: Yes

Reviewer #3: Yes

**********

3. Has the statistical analysis been performed appropriately and rigorously?

Reviewer #1: Yes

Reviewer #3: Yes

**********

4. Have the authors made all data underlying the findings in their manuscript fully available?

The PLOS Data policy

Reviewer #1: Yes

Reviewer #3: No

**********

5. Is the manuscript presented in an intelligible fashion and written in standard English?

Reviewer #1: Yes

Reviewer #3: Yes

**********

Reviewer #1: Thank you for addressing all the comments. However, minor english proof reading is necessary before final publications.

Reviewer #3: I would like to thank the authors for addressing reviewers' suggestions and improving the manuscript.

**********

what does this mean?). If published, this will include your full peer review and any attached files.

If you choose “no”, your identity will remain anonymous but your review may still be made public.

**Do you want your identity to be public for this peer review?**  For information about this choice, including consent withdrawal, please see our Privacy Policy

Reviewer #1: No

Reviewer #3: No

**********

# Editor report: Acceptance letter

**DOI:** 10.1371/journal.pone.0354954.r004

**Authors:** Claudia Noemi González Brambila

PONE-D-25-50366R1

PLOS One

Dear Dr. Vázquez,

I'm pleased to inform you that your manuscript has been deemed suitable for publication in PLOS One. Congratulations! Your manuscript is now being handed over to our production team.

At this stage, our production department will prepare your paper for publication. This includes ensuring the following:

* All references, tables, and figures are properly cited

* All relevant supporting information is included in the manuscript submission,

* There are no issues that prevent the paper from being properly typeset

You will receive further instructions from the production team, including instructions on how to review your proof when it is ready. Please keep in mind that we are working through a large volume of accepted articles, so please give us a few days to review your paper and let you know the next and final steps.

Lastly, if your institution or institutions have a press office, please let them know about your upcoming paper now to help maximize its impact. If they'll be preparing press materials, please inform our press team within the next 48 hours. Your manuscript will remain under strict press embargo until 2 pm Eastern Time on the date of publication. For more information, please contact onepress@plos.org.

You will receive an invoice from PLOS for your publication fee after your manuscript has reached the completed accept phase. If you receive an email requesting payment before acceptance or for any other service, this may be a phishing scheme. Learn how to identify phishing emails and protect your accounts at https://explore.plos.org/phishing.

If we can help with anything else, please email us at customercare@plos.org.

Thank you for submitting your work to PLOS One and supporting open access.

Kind regards,

PLOS ONE Editorial Office Staff

on behalf of

Dr. Claudia Noemi González Brambila

Academic Editor

PLOS One
